# Mechanosensation of rice root border cells activates serotonin-mediated pre-invasion behaviors in *Meloidogyne graminicola* via DEG/ENaC channels

**DOI:** 10.1186/s12870-026-09616-1

**Published:** 2026-07-25

**Authors:** Xiping Long, Wenxuan Xiao, Xuqi Shi, Shan Ye, Deliang Peng, Zhong Ding, Zhuhong Yang

**Affiliations:** 1 College of Plant Protection, Hunan Agricultural University, Changsha, 410128 P.R. China; 2 Hunan Provincial Engineering and Technology Research Center for Biopesticide and Formulation Processing, Changsha, 410128 P.R. China; 3 State Key Laboratory for Biology of Plant Diseases and Insect Pests, Institute of Plant Protection, Chinese Academy of Agricultural Sciences, Beijing, 100193 P.R. China

**Keywords:** Mechanosensation, Root border cells, Cellulose, DEG/ENaC channel, Serotonin, Stylet thrusting, *Meloidogyne graminicola*

## Abstract

**Background:**

Plant-parasitic nematodes (PPNs) locate host roots primarily through chemosensation, yet the sensory events that convert root contact into the stylet thrusts required for host penetration remain obscure. Root border cells (RBCs), terminally differentiated cells shed from the root cap, constitute the first point of physical contact between the root and invading nematodes, but whether and how RBCs trigger pre-invasion behaviors is unknown.

**Results:**

Here we showed that rice (*Oryza sativa*) RBCs induce preparasitic second-stage juveniles (pre-J2) of the rice root-knot nematode *Meloidogyne graminicola* to exhibit stylet thrusting, feeding behaviors, and marked up‑regulation of the serotonin biosynthesis gene *Mg‑tph‑1* in a density‑dependent manner. Among RBC components, the insoluble polysaccharide cellulose is the predominant inducer. We identified two Degenerin/Epithelial Sodium Channel (DEG/ENaC) mechanosensory genes, *Mg‑del‑1* and *Mg‑unc‑105*, both highly expressed in infective J2. RNA interference targeting either gene reduced nematode motility and abolished RBC‑ or cellulose‑induced stylet thrusting and feeding. Furthermore, silencing *Mg‑del‑1* or *Mg‑unc‑105* downregulated the serotonergic pathway genes *Mg‑unc‑43* and *Mg‑tph‑1*, and markedly reduced 5‑HT immunoreactivity in J2 responding to mechanical stimulation.

**Conclusions:**

We propose that specific DEG/ENaC channels mediate the mechanosensing of RBCs or root cellulose by root‑knot nematodes, activating a downstream serotonergic circuit that drives stylet thrusting. This work uncovers a previously uncharacterized mechanosensory pathway in plant‑nematode interactions and identifies nematode DEG/ENaC channels as promising targets for disrupting parasitism, offering a new mechanosensory‑based strategy for nematode control.

**Supplementary Information:**

The online version contains supplementary material available at https://doi.org/10.1186/s12870-026-09616-1.

## Background

Root-knot nematodes (*Meloidogyne* spp.) constitute one of the most economically damaging groups of plant-parasitic nematodes (PPNs), causing billions of dollars in annual crop losses worldwide [1]. Among them, *Meloidogyne graminicola* has emerged as a major threat to rice production in South and Southeast Asia, where this nematode is particularly well adapted to the flooded conditions characteristic of paddy ecosystems [2]. The success of *M. graminicola* as a rice parasite depends on its ability to locate host roots, penetrate root tissues, and establish functional feeding sites. During the soil-dwelling stage, preparasitic second-stage juveniles (pre-J2) must locate and invade host roots within a limited timeframe to obtain nutrients and complete their lifecycle. Upon reaching the root surface, the nematode typically slides past the root cap and penetrates the plant cell wall in the elongation zone, just behind the border cell region. At this site, the nematode initiates a series of precisely coordinated pre-invasion behaviors, culminating in rhythmic stylet thrusting that breaches the plant cell wall and allows entry into the host. Whereas long-range host-seeking by PPNs is largely governed by chemosensation of root exudates [3, 4], the sensory transition from host location to actual tissue penetration, particularly how nematodes perceive root contact and convert this tactile input into stylet activity, has remained poorly understood.

A critical interface between the root and its surrounding environment is formed by the root border cells (RBCs), terminally differentiated cells continuously shed from the root cap into the rhizosphere during root growth [5]. These living cells, embedded in a mucilaginous matrix rich in polysaccharides, glycoproteins, and extracellular DNA, constitute the root extracellular trap (RET)—a dynamic defense network that acts as the first physical and biochemical barrier against soilborne pathogens [6]. The RBC-mucilage complex facilitates interactions with both beneficial and pathogenic microorganisms, with border cells exerting dual functions as both a living protective barrier and a signaling hub in the rhizosphere [5]. In the context of plant-nematode interactions, RBCs are not merely passive obstacles but actively influence nematode behavior. In maize, RBCs enhance the motility of *M. incognita* upon contact without exerting strong chemotactic attraction [7]; in pea, border cells attract nematodes and subsequently induce a quiescent state [8]. These species-dependent responses suggest that RBC-derived signals can affect the behavior of nematodes. However, the nature of these signals—whether primarily mechanical cues or chemical signals—remains to be elucidated.

Mechanosensation, the ability to detect mechanical forces, is essential for diverse sensory functions including touch, hearing, nociception, and proprioception [9]. Mechanical stimuli induce conformational changes in mechanosensitive channel proteins, converting physical force into electrochemical signals [10–12]. In the model organism *Caenorhabditis elegans*, the degenerin/epithelial sodium channels (DEG/ENaC) family has been extensively characterized as the major mechanotransduction pathway [13]. DEG/ENaC subunits assemble into trimeric cation channels that are activated by membrane tension and extracellular mechanical forces [14]. The genetic tractability of *C. elegans* has enabled the functional dissection of specific DEG/ENaC roles: MEC-4 and MEC-10 form the core touch transduction channel in sensory neurons [15, 16], whereas DEGT-1 functions as a proprioceptor that monitors pharyngeal movement against the basement membrane to regulate feeding rate [17, 18]. Recently, nearly a quarter of neurons along the mammalian gastrointestinal tract were found to encode mechanosensory information, underscoring the fundamental importance of mechanical sensing in digestive function [19]. PPNs share conserved DEG/ENaC family members with their free-living counterparts, raising the compelling possibility that these channels have been co-opted during evolution to detect plant-derived mechanical cues, thereby linking root contact to the initiation of parasitic behaviors.

In this study, we found that rice RBCs trigger pre‑invasion behaviors in *M. graminicola* via a mechanosensory pathway. RBCs exposure induces stylet thrusting, feeding, and upregulation of the serotonin synthesis gene *Mg‑tph‑1* in a density‑dependent manner. Among RBCs components, cellulose is the most potent inducer. We identified two DEG/ENaC mechanosensory channel genes, *Mg‑del‑1* and *Mg‑unc‑105*, expressed in infective juveniles. Silencing either gene reduces nematode motility, abolishes RBC‑ and cellulose‑induced stylet thrusting, and decreases feeding. Silencing of these genes also reduces expression of serotonergic pathway genes and 5‑HT immunoreactivity. We propose that root border cells or root cellulose trigger a serotonergic circuit via DEG/ENaC-mediated mechanotransduction, thereby driving stylet thrusting. This work highlights mechanosensation as a key regulator of early parasitism in *M. graminicola* and positions DEG/ENaC channels as promising targets for disrupting nematode invasion.

## Materials and methods

### Nematode and plant materials

The nematode *M. graminicola* was isolated from a paddy field in Pingjiang County, Hunan Province (28°42′N, 113°35′E) and inoculated onto susceptible rice plants (*cv*. Nipponbare) for reproduction [20]. Pre-J2 and parasitic stage nematodes were collected as previously described [21].

The rice (*Oryza sativa*) seeds of varieties Huanghuazhan, IR64, Nongken58, Nipponbare, and Zhonghua11 were kindly provided by Professor Huafeng Deng at the Hunan Hybrid Rice Research Center (Changsha, Hunan, China). The seeds of variety Huaidao5 were kindly provided by Professor Lihui Wei at the Institute of Plant Protection, Jiangsu Academy of Agricultural Sciences (Nanjing, Jiangsu, China).

### Extraction of exudates, border cells, and tissue extracts from rice root tips

Rice seeds were surface-sterilized in 5% NaClO for 20 min, rinsed three times with sterile water, and germinated on moist filter paper at 30 °C for 48 h. Germinated seeds were transferred to a 96-well hydroponic box containing sterile water and cultivated in a growth chamber (14/10 h light/dark, 25 °C) for 5–7 days without changing the water.

A 5-mm segment was selected because the root apical meristem and the root border cell zone are concentrated within the first 3–5 mm of the rice root tip [22, 23]; this length reliably captures both the border cell population and the elongation zone, where nematode penetration typically occurs.

Root exudates were collected by immersing the 5-mm root tip of seedlings in 1 mL of sterile water in a 2 mL tube, gently shaken for 10 s, and then centrifuged at 500 ×g for 5 min. The supernatant was collected as crude root exudates, concentrated by freeze-drying, and used immediately.

RBCs were isolated by excising 5-mm root tip segments, vortexing in 200 µL sterile water for 60 s, and washing twice with 1 mL sterile water followed by centrifugation at 500 ×g for 3 min. The pellet was resuspended in sterile water to the desired density for immediate use.

Root tip tissue extracts were prepared by grinding 5-mm root tip segments in liquid nitrogen, homogenizing in PBS (137 mM NaCl, 2.7 mM KCl, 10 mM Na₂HPO₄, 1.8 mM KH₂PO₄, pH 7.4) containing a complete EDTA-free protease inhibitor cocktail (Roche, 11873580001), and centrifuging at 12,000 ×g for 15 min at 4 °C. The supernatant was collected as tissue extract and used immediately.

### Analysis of stylet thrusting frequency in J2 treated with root-derived components

The stylet thrusting frequency of J2 was assessed using a modified protocol based on a previously described method [20]. In brief, freshly hatched J2 were treated with the prepared root exudates, RBCs suspensions, or root tip tissue extracts for 1 h at 25 °C. Following treatment, J2 were randomly transferred to a glass slide. The number of stylet thrusts per nematode was recorded under a microscope over a 1-minute period, and the thrusting frequency (thrusts per minute) was calculated for each individual. For each treatment, at least 15 nematodes were observed across three independent replicates.

### Analysis of gene expression by qRT-PCR

Gene expression levels were analyzed by quantitative reverse transcription PCR (qRT-PCR). Treated nematodes were collected, washed three times with RNase-free water, and pelleted by centrifugation. Total mRNA was isolated using the Dynabeads™ mRNA DIRECT™ Kit (Invitrogen), and first-strand cDNA was synthesized with a PrimeScript RT reagent kit (TaKaRa). qRT-PCR was performed with gene-specific primers (see Supplementary Table 1) using TB Green Premix (TaKaRa) on a real-time PCR system. The *Mg-Actin2* gene served as the internal reference for normalization [24]. Relative gene expression was calculated using the 2^⁻∆∆Ct^ method [25]. Primer efficiencies ranged from 95% to 105%, as calculated from standard curves generated by 10-fold serial dilutions of cDNA. Melting curve analysis was performed at the end of each run to confirm the amplification of a single specific product. The qPCR cycling conditions were as follows: initial denaturation at 95 °C for 30 s, followed by 40 cycles of 95 °C for 5 s and 60 °C for 30 s, with a melting curve analysis step of 95 °C for 15 s, 60 °C for 60 s, and 95 °C for 15 s. Approximately 1,000 J2 were used for RNA extraction per biological replicate, and each treatment included at least three biological replicates. Each qPCR reaction was run in triplicate technical replicates. Statistical significance was determined using one-way ANOVA followed by Duncan’s multiple range test (*p* < 0.05).

### Assessment of root border cell number and viability

RBCs were isolated from the primary roots of rice varieties, including susceptible indica (Huanghuazhan, IR64), susceptible japonica (Nongken58, Nipponbare), and resistant japonica (Zhonghua11, Huaidao5). For each variety, 5-mm root tip segments were collected from roots of varying lengths (5 to 80 mm). The segments were vortexed in deionized water for 60 s to release RBCs. Cell viability was determined using the AO-EB Double Fluorescence Staining Kit (MKBio, China) following the manufacturer‘s instructions. The RBCs suspension was briefly stained and then loaded onto a hemocytometer. The total number of RBCs, along with viable (green fluorescence) and dead (red fluorescence) cells, were counted under a fluorescence microscope. Cell viability was calculated as (number of viable cells / total number of cells) × 100%. Each measurement was performed with three replicates.

### In situ hybridization analysis

For in situ hybridization, 10,000 pre-J2 *M. graminicola* were collected. J2 were fixed in 4% paraformaldehyde, permeabilized with proteinase K, and post-fixed. Digoxigenin (DIG)-labeled sense and antisense RNA probes were synthesized using gene-specific primers (Supplementary Table 1) with T7 RNA polymerase (Roche, 53760220). Hybridization was carried out overnight at 55 °C in hybridization buffer (50% formamide, 5× SSC, 1% blocking reagent, 0.1% Tween-20). After stringent washes, the hybridized probes were detected using an anti-DIG-alkaline phosphatase antibody (1:2000, Roche) and NBT/BCIP substrate. The stained nematodes were mounted and imaged under a light microscope.

### Cy3 staining

The uptake activity of J2 was assessed using the fluorescent dye cyanine 3 (Cy3, UElandy, C5080) based on a previously described method [26] with modifications. Briefly, freshly hatched J2 were treated with RBCs suspensions at various concentrations for 1 h at 25 °C. After treatment, the nematodes were incubated in 0.2 mg/mL Cy3 solution for 5 min. Subsequently, approximately twenty nematodes from each treatment were collected, rinsed three times with sterile water to remove excess dye, and transferred onto a glass slide. The samples were heat-killed and immediately observed under the inverted fluorescence microscope Zeiss Axio Observer 7 (Carl Zeiss AG, Oberkochen, Germany) using a suitable filter set for Cy3 detection [20]. Fluorescence intensity within the nematode esophagus was quantified using ImageJ software. For each treatment, the experiment was performed with at least three independent replicates.

### Tissue sectioning of rice root tips

For histological investigation, the paraffin-embedding method was followed [27]. Briefly, root tips from IR64 and Nipponbare rice cultivars were fixed in 8% paraformaldehyde at 4 °C. Following dehydration in a graded ethanol series, the tissues were infiltrated with methyl methacrylate-based resins, embedded, and polymerized under UV light. For each rice cultivar, five root tips were analyzed, and from each root tip, at least 15 Sect. (8 μm thick) were examined to ensure representative sampling. Section (8 μm thick) were cut, dried, and stained with 0.1% toluidine blue. After dehydration, the sections were mounted in PBS (137 mM NaCl, 2.7 mM KCl, 10 mM Na₂HPO₄, 1.8 mM KH₂PO₄, pH 7.4) and imaged under a light microscope.

### Chemotaxis assay

The chemotactic response of J2 to RBCs from the rice cultivar Nipponbare was evaluated at densities of 3 × 10¹, 3 × 10², 3 × 10³, 3 × 10⁴, and 3 × 10⁵ RBCs/mL. In a chemotaxis assay plate (7.5 cm diameter), 10 µL of the RBCs suspension was added to sample well (a depression in the PF-127 gel prepared using a 200 µL pipette tip), while 10 µL of sterile water (control) was added to another. The plate was covered and kept at room temperature for 40 min to allow a chemical gradient to form within the 23% (w/v) Pluronic F-127 gel (Sigma-Aldrich, Catalog No. P2443). Subsequently, approximately 100 J2 were placed at the center of the plate. After incubation at room temperature for 4 h, the number of nematodes within a 0.5 cm radius of each sample well was counted under a microscope. The chemotaxis index (CI) was calculated as: CI = (Number of J2 in treatment sample well - Number of J2 in control sample well) / Total number of J2 in the assay. Each treatment was replicated three times, and the entire experiment was repeated at least three times.

### Immunohistochemical analysis of *M. graminicola* J2

After treatment with RBCs suspensions, J2 were processed for serotonin (5-HT, 5-hydroxytryptamine) immunohistochemistry as previously [20]. Briefly, nematodes were fixed in 4% formaldehyde, permeabilized with proteinase K following physical segmentation, and dehydrated through a graded series of methanol and acetone steps. After blocking with 1% BSA, samples were incubated overnight at 4 °C with a rabbit anti-serotonin primary antibody (1:100), followed by incubation with an FITC-conjugated goat anti-rabbit secondary antibody (1:200; Sigma‒Aldrich, F9887). Following final washes, the samples were mounted and imaged using a Zeiss LSM confocal microscope. This protocol enabled visualization of 5-HT localization and quantitation of serotonergic neuron activation in response to RBCs stimulation.

### Analysis of sugar components on nematode behavior

To investigate the influence of sugar components in the root tip microenvironment on *M. graminicola* behavior, we first determined the major monosaccharide composition of RBCs from susceptible indica (IR64) and japonica (Nipponbare) rice cultivars (profiled by targeted metabolomics, Sanshu Biotechnology). Subsequently, representative sugars—including monosaccharides (xylose, arabinose, galactose, glucose), disaccharides (sucrose, maltose), and polysaccharides (cellulose, arabinogalactan, pectin)—were systematically evaluated at concentrations of 2 µg/mL and 20 µg/mL for their effects on nematode stylet thrusting, feeding behavior, and chemotaxis.

### Cloning of *M. graminicola* mechanosensory genes

To clone target mechanosensory genes from *M. graminicola*, the following protocol was established. Approximately 10,000 nematodes were collected for total RNA extraction using Trizol reagent (Sangon Biotech). RNA quality and concentration were assessed with a spectrophotometer (Thermo), and integrity was confirmed by 1% agarose gel electrophoresis. First-strand cDNA was synthesized from the total RNA using the PrimeScript™ III First-Strand Synthesis System (Invitrogen) and served as the template for subsequent PCR and RACE (Rapid Amplification of cDNA Ends) reactions.

The putative coding sequence of the target gene was initially identified by performing a homology search in the WormBase Parasite database, using known mechanosensory *deg-1* (Gene ID: 181035), *del-1* (degenerin-like 1, Gene ID: 181534), *del-10* (Gene ID: 174069), *mec-4* (Gene ID: 181728), *mec-10* (Gene ID: 181101), *degt-1* (Gene ID: 184921) and *acd-1* (Gene ID: 182852) gene sequences from *C. elegans* as queries. To obtain the full-length cDNA, gene-specific primers were designed based on the predicted sequence (see Supplementary Table 1 for primer sequences used in this study). The 5’ end of the transcript was amplified by PCR using a universal spliced leader sequence (SL1) primer paired with a gene-specific reverse primer. The 3’ end was obtained using a 3’ RACE system (TaKaRa) according to the manufacturer’s instructions. Finally, the complete open reading frame (ORF) was amplified using primers designed within the confirmed untranslated regions.

### Bioinformatic analysis

For the bioinformatic analysis of candidate mechanosensory genes, the following procedures were performed. The full-length cDNA sequence was assembled using DNAMAN software (v9.1; Lynnon BioSoft), and the corresponding amino acid sequence was deduced. Conserved domains of the predicted protein were identified using the SMART database (http://smart.embl-heidelberg.de/) and the Conserved Domain Database (CDD) tool on the NCBI website (https://www.ncbi.nlm.nih.gov/Structure/cdd/), with a focus on regions associated with mechanosensation, such as transmembrane domains and pore loops. Multiple sequence alignment was conducted using ClustalW software (http://www.clustal.org/clustal2/), comparing the sequence with known mechanosensitive channel proteins from *C. elegans* and other species to assess the conservation of key residues. A phylogenetic tree was constructed with MEGA 7.0 using the Maximum Likelihood method with 1000 bootstrap replicates to determine the evolutionary position of the gene within the mechanosensitive channel family.

### In vitro RNAi targeting mechanosensory genes in *M. graminicola* J2

Total RNA was extracted from *M. graminicola* using TRIzol reagent, and first‑strand cDNA was synthesized. Gene‑specific primers flanked by the T7 promoter sequence (see Supplementary Table 1) were designed for each target mechanosensory gene. Double‑stranded RNAs (dsRNAs) were synthesized using the T7 RiboMAX™ Express RNAi System (Promega). The dsRNA fragments were designed as follows: for *Mg-del-1*, a 437 bp fragment targeting nucleotides 2204–2639 of the ORF; for *Mg-unc-105*, a 334 bp fragment targeting nucleotides 2221–2554 of the ORF. As a negative control, dsRNA targeting the green fluorescent protein (*gfp*) gene was prepared in parallel and dissolved in the same soaking buffer at the same concentration (2 mg/mL) as the target gene dsRNAs. An additional control group was treated with soaking buffer only (no dsRNA). Approximately 10,000 freshly hatched J2 were incubated in soaking buffer (0.5% glucose, 0.005% gelatin, 1% PEG 6000, 20 mM NaCl, 3 mM KCl, 0.7 mM MgSO₄, and 1 mM CaCl₂ in DEPC-treated water, pH 6.5). No neuromodulators (e.g., octopamine or spermidine) were added to the soaking buffer. The soaking buffer was then supplemented with 2 mg/mL of the target dsRNA (or *gfp* dsRNA for the control) and incubated at room temperature on a gentle rotator for 24 h. An additional control group was treated with soaking buffer only (no dsRNA). For each RNAi treatment, three independent biological replicates were performed. For RNA extraction and gene expression analysis, approximately 1,000 J2 were used per replicate; for phenotypic assays, approximately 100 J2 per replicate were used. After incubation, nematodes were washed three times with DEPC‑treated water. The treated J2 were either snap‑frozen and stored at − 80 °C for subsequent gene‑expression analysis or transferred to 1.5 mL centrifuge tubes for phenotypic assays.

### Motility assay for mechanosensory gene RNAi in *M. graminicola* J2

A sand column migration assay was performed to evaluate the motility of J2 following RNAi targeting of mechanosensory genes, as adapted from a previously described method [28, 29]. Briefly, approximately 100 J2 from each treatment group (target gene RNAi, *gfp* RNAi control, and buffer-only control) were transferred into PVC tubes (custom-made, 5 cm length, 1.5 cm internal diameter) packed with moist sand, with the bottom sealed using 150‑mesh nylon gauze (0.106 mm aperture). The tubes were then placed into 50 mm Petri dishes containing 20 mL of double‑distilled water (ddH₂O), ensuring the bottom of the sand column was submerged. The setup was incubated at 25 °C in darkness. After 6 h and 24 h, the number of nematodes that had migrated through the sand column into the Petri dish was recorded. The passage rate was calculated as follows:

$$\begin{aligned} \text{Passage rate}\left(\%\right)&=\left(\text{Number of nematodes}\:\right.\\&\left. \mathrm{migrated}/100\right)\times 100\% \end{aligned}$$

This assay allowed for quantitative comparison of locomotion ability among different RNAi treatment groups, providing a phenotypic measure related to mechanosensory function.

### Statistical analysis

All statistical analyses were conducted using SPSS software (version 17.0). Significant differences among multiple experimental groups were determined by one-way analysis of variance (ANOVA), followed by post-hoc comparisons using Duncan’s multiple range test. Differences were considered statistically significant when *p* < 0.05.

## Results

### Effect of rice RBCs on *M. graminicola* behavior

In our previous study, we found that nematode contact with root tips strongly induces the expression of *Mg-tph-1*, a gene encoding tryptophan hydroxylase, the rate-limiting enzyme in serotonin biosynthesis [20]. Given that PPNs initiate root invasion by puncturing epidermal cells with their stylets, we sought to identify the specific root-tip-derived factors that trigger this stylet activity. To this end, we extracted root exudates, RBCs, and root tissue extracts from the variety Nipponbare. We found that RBCs effectively stimulated *Mg-tph-1* expression and stylet thrusting (Supplementary Fig. 1; Supplementary movie 1).

Statistical analysis of RBC numbers across six rice varieties (Huanghuazhan, IR64, Nongken58, Nipponbare, Zhonghua11 and Huaidao5) revealed that RBC numbers followed a similar dynamic pattern of an initial increase followed by a decrease, with peak counts occurring at a root length of 35–40 mm, and that RBCs viability remained consistently high (> 88.9%) across all varieties (Supplementary Fig. 2). Given the conserved high viability and consistent peak abundance of RBCs across rice varieties, we next asked whether the density of RBCs encountered by *M. graminicola* at the root tip quantitatively modulates the nematode’s pre‑invasion behavioral and molecular responses.

**Fig. 1:**
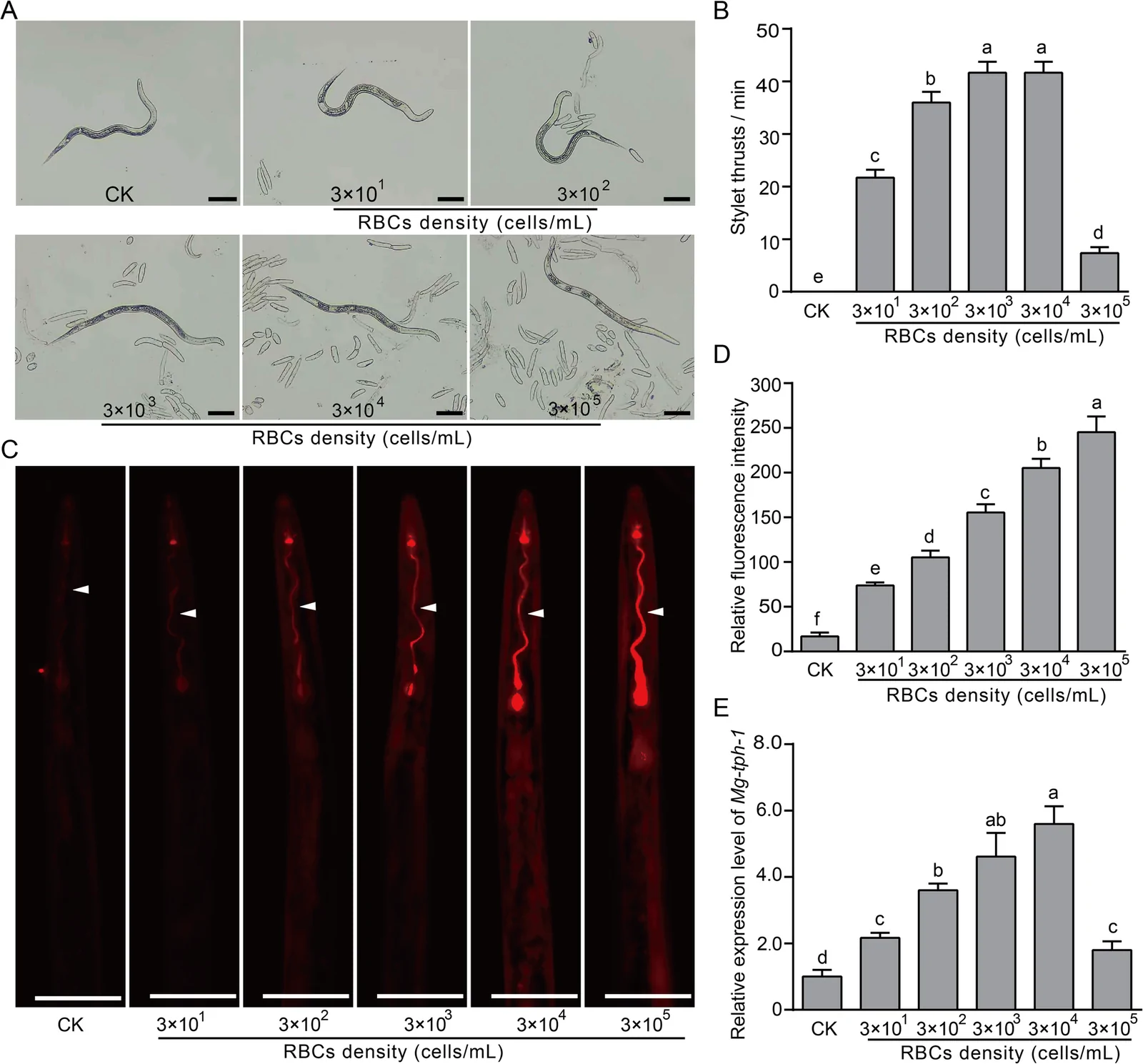
Rice root border cells (RBCs) induce *M. graminicola* preparasitic second-stage juveniles (pre-J2) stylet thrusting and feeding activity. **A** Schematic illustration of the experimental design: pre-J2 were exposed to RBCs suspensions at densities ranging from 3 × 10¹ to 3 × 10⁵ cells/mL for 1 h at 25 °C, with sterile water as the negative control (CK). **B** Stylet thrusting frequency recorded microscopically over a 1‑min observation period. **C** Representative fluorescence micrographs of Cy3 dye uptake in the esophageal region, indicating pharyngeal feeding activity. **D** Quantification of relative Cy3 fluorescence intensity in the esophageal region. **E** Relative expression level of *Mg‑tph‑1* determined by qRT‑PCR. All data are presented as mean ± standard deviations (SDs) from three independent biological replicates, with each replicate observing at least 15 nematodes per treatment. Different lowercase letters above the bars indicate statistically significant differences among treatments (one‑way ANOVA followed by Duncan’s multiple range test, *p* < 0.05). Bar = 50 μm

To further examine whether stylet activity depends on RBC density, we exposed *M. graminicola* J2 to Nipponbare RBCs at densities ranging from 3 × 10¹ to 3 × 10⁵ RBCs/mL (Fig. 1A). Stylet thrusting was observed at all densities tested, whereas no stylet activity was observed in the sterile water control group (Fig. 1B). The frequency of stylet thrusting increased with RBCs density from 3 × 10¹ to 3 × 10³ RBCs/mL, reaching a maximum average of 41.7 thrusts per minute at 3 × 10³ and 3 × 10⁴ RBCs/mL, with no significant difference between these two groups. However, when the RBCs density was further increased to 3 × 10⁵ RBCs/mL, the average thrusting frequency decreased significantly to 7.3 times per minute.

Following exposure to RBCs, distinct Cy3 fluorescence signals were detected in the esophageal region of the nematodes, whereas no detectable fluorescence was observed in this region of nematodes treated with sterile water (Fig. 1C-D). In the low‑density treatment groups (3 × 10¹–3 × 10³ RBCs/mL), the relative fluorescence intensity was increased by 4.1‑ to 5.5‑fold compared with the background signal of the control group. Increasing the RBCs density to 3 × 10⁴ RBCs/mL elevated the relative fluorescence intensity to 8.8‑fold that of the control. At the highest density tested (3 × 10⁵ RBCs/mL), fluorescence intensity reached 9.3‑fold that of the control. These results demonstrate that higher RBCs densities enhance tracer accumulation in the esophageal region, consistent with increased uptake activity of the nematodes.

We quantified *Mg-tph-1* expression by qRT-PCR in nematodes exposed to varying densities of Nipponbare RBCs (3 × 10¹ to 3 × 10⁵ RBCs/mL, Fig. 1E). *Mg-tph-1* expression increased in a density‑dependent manner, showing a 2.5‑ to 5.6‑fold upregulation at 3 × 10¹ to 3 × 10⁴ RBCs/mL compared with the sterile‑water control. Expression peaked at 3 × 10⁴ RBCs/mL, reaching a 5.6‑fold induction over the control, but then declined at 3 × 10⁵ RBCs/mL to a level comparable to that observed at 3 × 10¹ RBCs/mL (Fig. 1E). Immunohistochemical staining revealed the dynamic changes in serotonergic neuron activity of *M. graminicola* following exposure to Nipponbare RBCs at 3 × 10⁵ RBCs/mL for 1 h and 2 h. In untreated J2, two neuronal pairs were evident: ADF-like neurons and a second, unnamed pair located near the nerve ring. After 1 h of exposure, an additional RIH-like neuron appeared posterior to the ADF-like pair, and fluorescence intensity increased in the original pairs. After 2 h, an NSM-like neuron emerged in the median bulb region, while ADF-like and RIH-like neurons remained detectable (Supplementary Fig. 3A). Additionally, an agar composite gel assay across a gradient of RBC densities (3 × 10¹ to 3 × 10⁵ cells/mL) revealed that *M. graminicola* J2 exhibited no attraction at lower densities and only weak chemotactic activity at the highest density (3 × 10⁵ cells/mL), with a mean chemotaxis index (CI) of 0.15 (Supplementary Fig. 3B).

Collectively, these data demonstrated that RBCs function as critical signals that trigger pre‑invasion behaviors in *M. graminicola*. RBCs stimulate stylet thrusting, feeding activity, and upregulate *Mg‑tph‑1*. Nematode responses are dose‑dependent: low‑to‑medium densities (≤ 3 × 10⁴ cells/mL) enhance stylet activity and *Mg-tph-1* expression, whereas high density (3 × 10⁵ cells/mL) suppresses these responses while further elevating feeding activity. Immunohistochemical analysis indicates that RBCs exposure also activates and reconfigures serotonergic circuitry.

### Impact of carbohydrate on nematode behavior

RBCs originate from the root cap meristematic cells and form a specialized layer surrounding the root tip [30]. These cells occur as single cells or multicellular aggregates and are embedded within a mucilaginous matrix. In rice, RBCs are typically elongated and form adherent clusters [22, 23], which is consistent with our histological observation of longitudinal sections through the root apical meristem of susceptible cultivars IR64 and Nipponbare (Supplementary Fig. 4A). The RBCs cell wall is primarily composed of pectin and cellulose, while the mucilage contains a complex mixture of monosaccharides [31]. Targeted metabolomic analysis of RBCs from IR64 and Nipponbare further revealed the detailed monosaccharide profile: galactose (27.33–28.00%), arabinose (24.98–25.23%), xylose (18.61–19.90%), and glucose (11.20–12.29%) were the predominant components, with fucose, mannose, rhamnose, glucuronic acid, and galacturonic acid present in minor fractions (Supplementary Fig. 4B). As migrating nematodes encounter the root tip, they first contact the RBCs and the surrounding mucilage, which is enriched in high‑molecular‑weight polysaccharides.

**Fig. 2:**
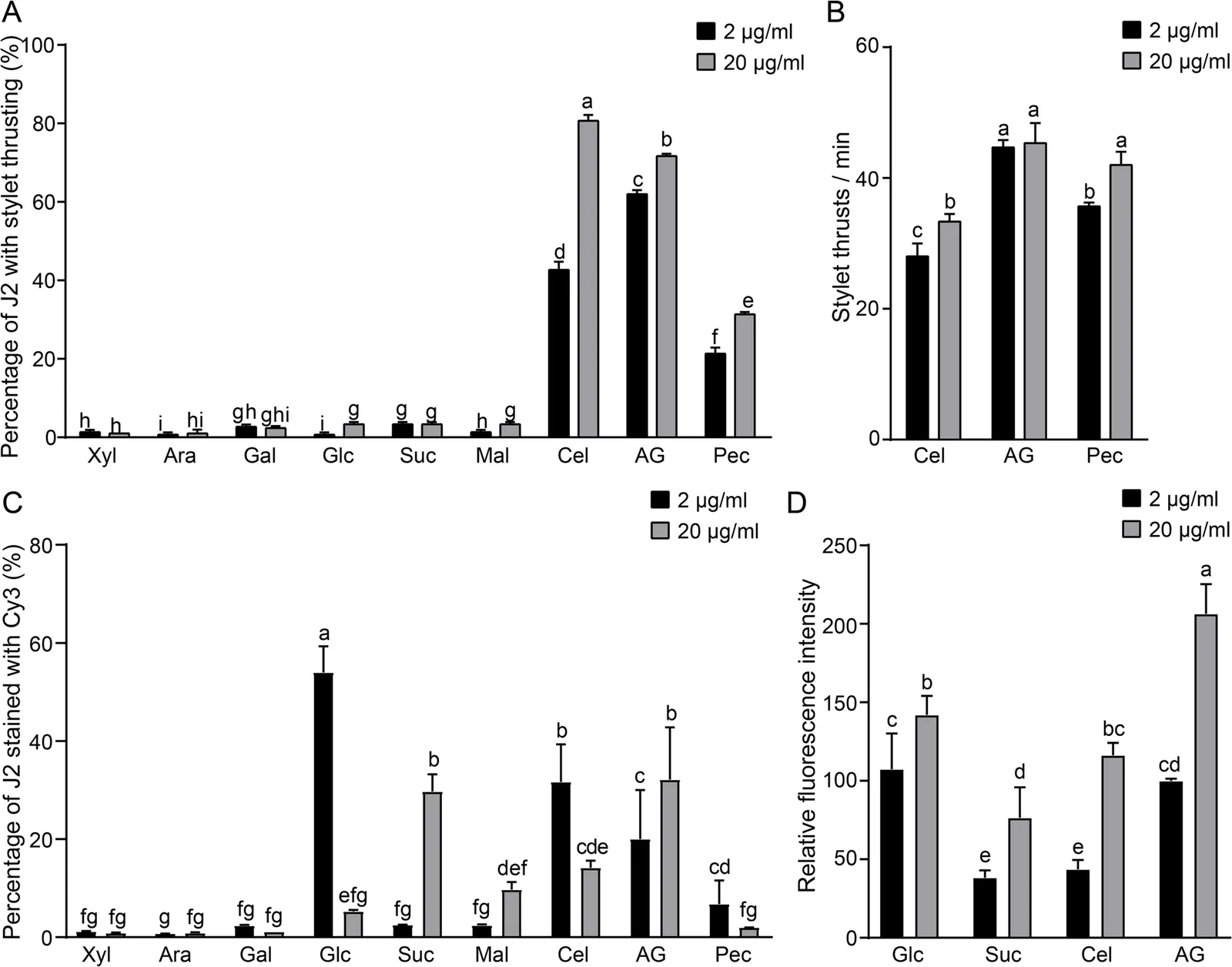
Effects of root tip sugars on *M. graminicola* pre-J2 feeding behavior. Pre‑ J2 were exposed for 1 h to representative monosaccharides (Xyl, xylose; Ara, arabinose; Gal, galactose; Glc, glucose), disaccharides (Suc, sucrose; Mal, maltose), or polysaccharides (Cel, cellulose; AG, arabinogalactan; Pec, pectin) at two concentrations (2 µg/mL and 20 µg/mL). Sterile water served as the negative control (not shown; no responses above background). Behavioral and feeding parameters were then quantified microscopically and by Cy3 dye uptake. **A** Percentage of J2 showing stylet thrusting in response to different sugars at 2 or 20 µg/mL. **B** Stylet thrust frequency of J2 treated with polysaccharides. **C** Percentage of J2 showing Cy3 dye uptake under sugar treatments. **D** Relative fluorescence intensity (Cy3 uptake) for sugars. All data are presented as mean ± standard deviations (SDs) from three independent experiments, with each experiment scoring at least 20 J2 per treatment (*n* = 20 per replicate). Different lowercase letters above the bars within each panel denote statistically significant differences among treatments (one‑way ANOVA followed by Duncan’s multiple range test, *p* < 0.05)

To determine whether specific carbohydrate components of the RBC wall and mucilage modulate nematode parasitic behavior, we evaluated the effect of representative sugars on stylet thrusting. J2 were exposed to solutions of monosaccharides (xylose, arabinose, galactose, glucose), disaccharides (sucrose, maltose), and polysaccharides (cellulose, arabinogalactan, pectin) at 2 µg/mL and 20 µg/mL. The proportion of J2 exhibiting stylet thrusting was microscopically observed and quantified. Monosaccharides and disaccharides elicited minimal responses, with the proportion of J2 showing stylet thrusting remaining below 3.4% at both concentrations. In contrast, polysaccharides induced a markedly higher proportion of J2 to exhibit stylet thrusting, and this response scaled with concentration. Cellulose elicited the strongest effect, with 42.7% and 80.7% of J2 exhibiting stylet thrusting at 2 µg/mL and 20 µg/mL, respectively. Arabinogalactan induced thrusting in 62.0% and 71.7% of J2 at 2 µg/mL and 20 µg/mL, respectively, while pectin induced lower rates of 21.3% and 31.3% at the corresponding concentrations (Fig. 2A). Consistently, analysis of thrusting frequency (Fig. 2B) showed significant, concentration‑dependent increases for all polysaccharides, exceeding 28.0 thrusts/min in all groups. Arabinogalactan at 20 µg/mL exerted the strongest stimulatory effect, with an average thrusting frequency of 45.3 thrusts/min.

We next evaluated nematode feeding behavior using a fluorescence-based uptake assay. All tested sugars at concentrations of 2 µg/mL and 20 µg/mL induced detectable fluorescence signals in the esophageal region of nematodes, whereas no signal was observed in the sterile‑water control group. Among monosaccharides (Fig. 2C), glucose uniquely stimulated feeding in a substantial fraction of nematodes at 2 µg/mL (54.0%), but this response declined sharply at 20 µg/mL (5.3%). Xylose, arabinose, and galactose elicited feeding in < 2.3% of J2. Among disaccharides, only sucrose induced feeding (29.7%) at 20 µg/mL. For polysaccharides, cellulose and pectin supported higher feeding at 2 µg/mL (31.7% and 14.5%, respectively) but showed reduced responses at 20 µg/mL (14.2% and 2.0%). In contrast, arabinogalactan promoted feeding in a concentration‑dependent manner (20.0% at 2 µg/mL, 32.1% at 20 µg/mL). Fluorescence intensity analysis of the sugars that elicited higher feeding rates—glucose, sucrose, cellulose, and arabinogalactan (Fig. 2D)—revealed that average fluorescence intensity increased with concentration for all four compounds. Arabinogalactan at 20 µg/mL showed the highest intensity (206.3). Glucose produced intensities of 107.3 at 2 µg/mL and 142.0 at 20 µg/mL, indicating a sustained feeding preference.

We further assessed the chemotactic activity of these sugars for *M. graminicola* J2 using an agar composite gel assay. Sucrose and arabinogalactan elicited no chemotactic response at either tested concentration, whereas glucose, maltose, cellulose and pectin induced weak chemotactic attraction (Supplementary Fig. 5A). At 2 µg/mL, glucose (CI = 0.12) and cellulose (CI = 0.11) exhibited significantly higher chemotaxis indices than the other tested sugars at the same concentration. At 20 µg/mL, the chemotaxis indices of these four sugars increased slightly to 0.13 (glucose), 0.16 (maltose), 0.16 (cellulose) and 0.14 (pectin), with no significant differences detected among them. Within the tested concentration range, only glucose and cellulose triggered weak, concentration-dependent chemotaxis in J2. Notably, cellulose treatment additionally induced prominent nematode aggregation (Supplementary Fig. 5B).

In summary, polysaccharides strongly induced stylet thrusting in *M. graminicola*, whereas monosaccharides and disaccharides showed minimal effects. The induction of stylet thrusting by polysaccharides suggests that both chemical and physical stimuli from root cells contribute to this behavioral response. Among the tested polysaccharides, arabinogalactan is water-soluble, whereas cellulose is insoluble. In the rhizosphere environment, soluble arabinogalactan may be rapidly diluted or washed away, limiting its availability as a sustained cue. In contrast, the insoluble cellulose fibrils are physically anchored in the root cell wall, providing a stable and persistent mechanical stimulus. Therefore, we propose that while both chemical and physical signals are involved, the insoluble cellulose acts as the predominant physical cue triggering stylet thrusting upon root contact. Notably, glucose uniquely stimulated nematode feeding at low concentrations. Despite weak chemotactic responses, cellulose also triggered nematode aggregation, highlighting its role as a key physical cue in root–nematode interactions.

### Identification and functional characterization of mechanosensory genes in *M. graminicola*

Based on the finding that cellulose (a major RBCs component) is the key mechanical cue, we further identified mechanosensory channels that mediate this signal transduction. Using the predicted sequences of *C. elegans* mechanosensory protein as queries, we performed BLAST searches against the *M. graminicola* genome and identified six highly homologous candidate genes: *Mgra_00002414* (GenBank accession KAF7638186.1), *Mgra_00005966* (KAF7634628.1), *Mgra_00002017* (KAF7638639.1), *Mgra_00003938* (KAF7636549.1), *Mgra_00000584* (KAF7640138.1), and *Mgra_00009194* (KAF7629299.1). Among these, two DEG/ENaC-type mechanosensory channel genes—*Mg-del-1* (from *Mgra_00005966*, GenBank accession PX755864) and *Mg-unc-105* (from *Mgra_00003938*, PX755863)—were cloned and functionally characterized. Full-length cDNAs were obtained using specific primers (Supplementary Table 1). *Mg‑del‑1* is 3085 bp in length and contains a 2950 bp ORF encoding a 981‑amino‑acid protein (109.35 kDa). *Mg‑unc‑105* spans 3226 bp cDNA, including a 2907 bp ORF for a 967‑amino‑acid protein (111.51 kDa).

Protein BLAST analysis against NCBI identified 45 representative degenerin family proteins from nematodes for phylogenetic analysis (Supplementary Fig. 6). Mg‑DEL‑1 shared 50.79% similarity with *C. elegans* DEL‑1 and 79.73% with *M. enterolobii* CAK5091297.1. Mg‑UNC‑105 clustered with *C. elegans* UNC‑105 (44.57% similarity) and shared 76.00% and 71.19% similarity to homologs from *M. hapla* (KAL7071408.1) and *M. enterolobii* (CAK5091060.1), respectively. Multiple sequence alignment confirmed that both proteins (Mg‑DEL‑1 and Mg‑UNC‑105) possess the conserved amiloride-sensitive sodium channel domain characteristic of the degenerin/ENaC channel (Supplementary Fig. 7–8).

**Fig. 3:**
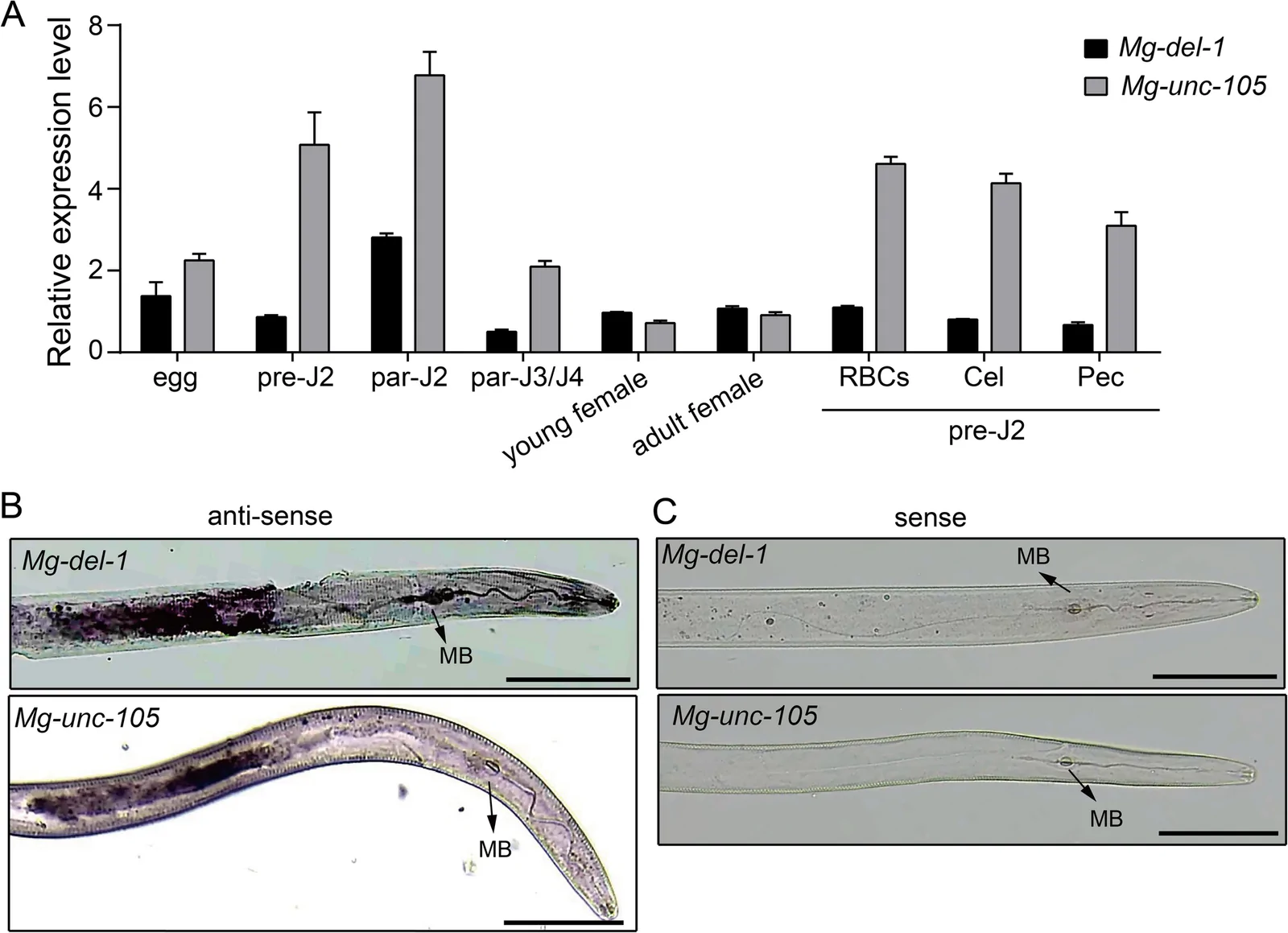
Expression and localization of the DEG/ENaC mechanosensory channel genes *Mg‑del‑1 *and* Mg‑unc‑105 *in* M. graminicola*. **A** Relative expression levels of *Mg-del-1* and *Mg-unc-105* in different life stages (egg; pre-J2, preparasitic second-stage juvenile; par-J2, parasitic second-stage juvenile; par-J3/J4, parasitic third/fourth-stage juvenile; young female; adult female) of *M. graminicola* and in J2 treated with root border cells (RBCs, 3 × 10⁴ cells/mL), cellulose (Cel, 20 µg/mL), or pectin (Pec, 20 µg/mL). **B** In situ hybridization localization of *Mg‑del‑1* and *Mg‑unc‑105* transcripts in pre‑J2 using digoxigenin‑labeled antisense RNA probes. **C** Control hybridizations performed with corresponding sense probes showed no specific staining, confirming the specificity of the antisense signals. Arrowheads indicate the median bulb (MB). Bar = 50 μm

Quantitative qRT-PCR analysis across developmental stages demonstrated that *Mg‑del‑1* transcripts peak in parasitic second‑stage juveniles (par‑J2), whereas *Mg‑unc‑105* was highly expressed in both pre‑J2 and par-J2 stages. However, treatment of pre‑J2 with RBCs, cellulose, or pectin revealed that neither of the two genes (*Mg‑del‑1* and *Mg‑unc‑105*) were induced by any of these treatments (Fig. 3A). Additionally, in situ hybridization was performed to localize the transcripts of *Mg-del-1* and *Mg-unc-105*. Both genes exhibited widespread expression with notably strong signals in the intestinal region, and a distinct and intense *Mg‑del‑1* signal was specifically observed in the median bulb (Fig. 3B). In contrast, *Mg‑unc‑105* showed only background‑level signal in this structure. Control hybridizations with sense probes showed no specific staining (Fig. 3C).

**Fig. 4:**
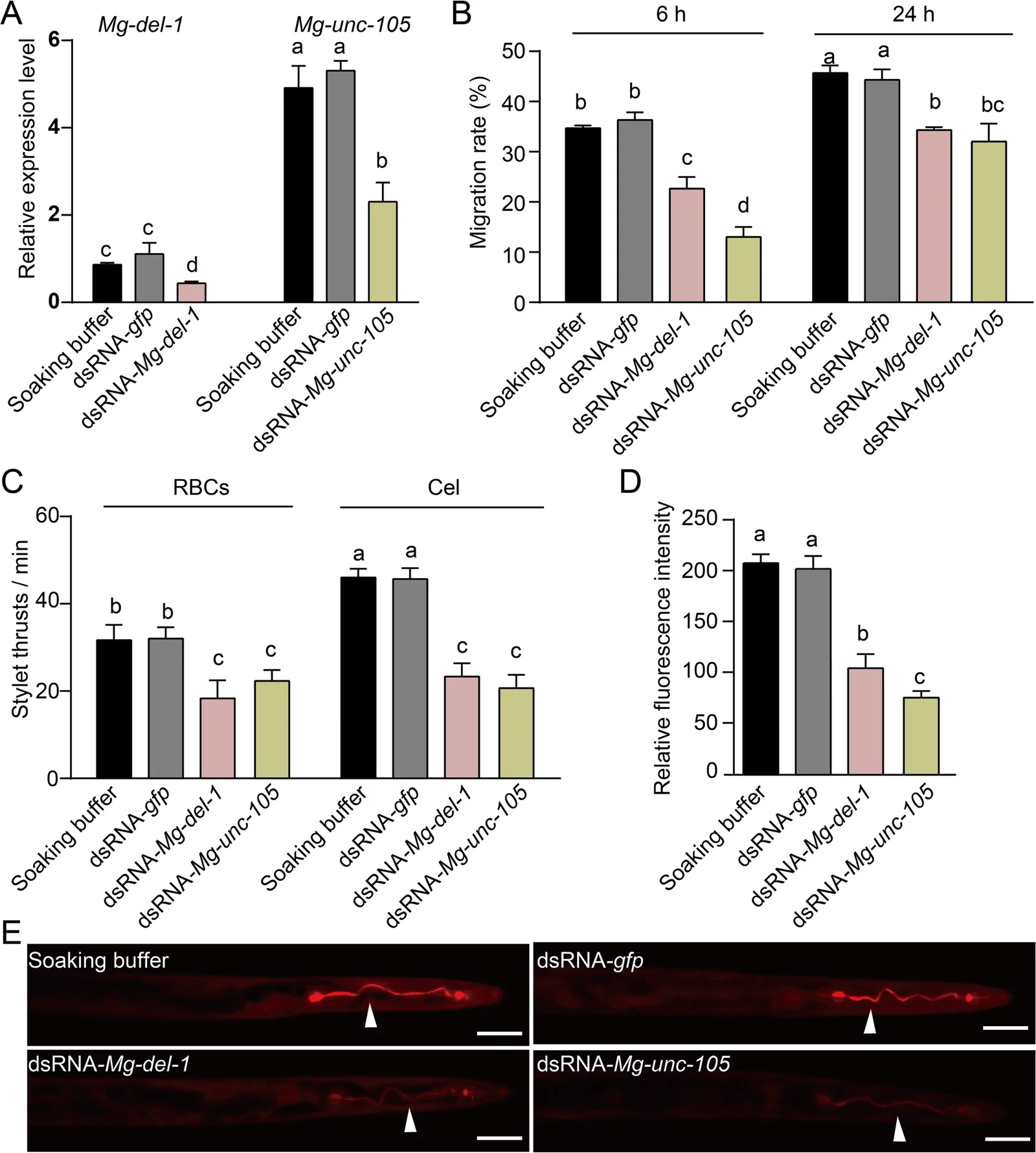
Effects of silencing mechanosensory genes on *M. graminicola* J2 motility, stylet thrusting, and feeding activity. **A** The relative expression level of *Mg-del-1* and *Mg-unc-105* genes after J2 were incubated in soaking buffer, target gene dsRNA or *gfp* dsRNA for 24 h. **B** Migration rate of J2 through a sand column assessed at 6 h and 24 h after inoculation. Approximately 100 J2 were pre-treated with the dsRNA targeting *Mg-del-1* or *Mg-unc-105* (dsRNA-gfp and soaking buffer served as controls). **C** Stylet thrusting frequency measured after exposing the dsRNA‑treated J2 to either root border cells (RBCs, 3 × 10⁴ cells/mL) or cellulose (Cel, 20 µg/mL) for 1 h. Fluorescence intensity (**D**) and distribution (**E**) of Cy3 fluorescence intensity in the esophageal region, reflecting pharyngeal feeding activity. Bar = 20 μm. All quantitative data are presented as mean ± standard deviations (SDs) pooled from three independent experiments, with n= 20 J2 scored per treatment per replicate. Different lowercase letters above the bars within each panel indicate statistically significant differences among treatments (one‑way ANOVA followed by Duncan’s multiple range test, *p* < 0.05)

To assess the role of these channels in nematode behavior, J2 were soaked in gene-specific dsRNA or controls for 24 h. Efficient silencing was confirmed by qRT-PCR (Fig. 4A). In the sand column migration assay, control and dsRNA-*gfp* groups exhibited 34.6% and 36.3% migration at 6 h, respectively, whereas the dsRNA‑*Mg‑del‑1* and dsRNA‑*Mg‑unc‑105* groups showed significantly reduced migration rates of 22.6% and 13.0%, respectively (Fig. 4B). These reductions persisted at 24 h. Similarly, stylet thrusting assays after RBCs or cellulose exposure revealed significant decreases in thrust frequency in all dsRNA-treated groups relative to controls (Fig. 4C). Feeding assays, monitored by Cy3 dye uptake, showed that *Mg-del-1* and *Mg-unc-105* silencing impaired ingestion under cellulose stimulation (Fig. 4D–E).

**Fig. 5:**
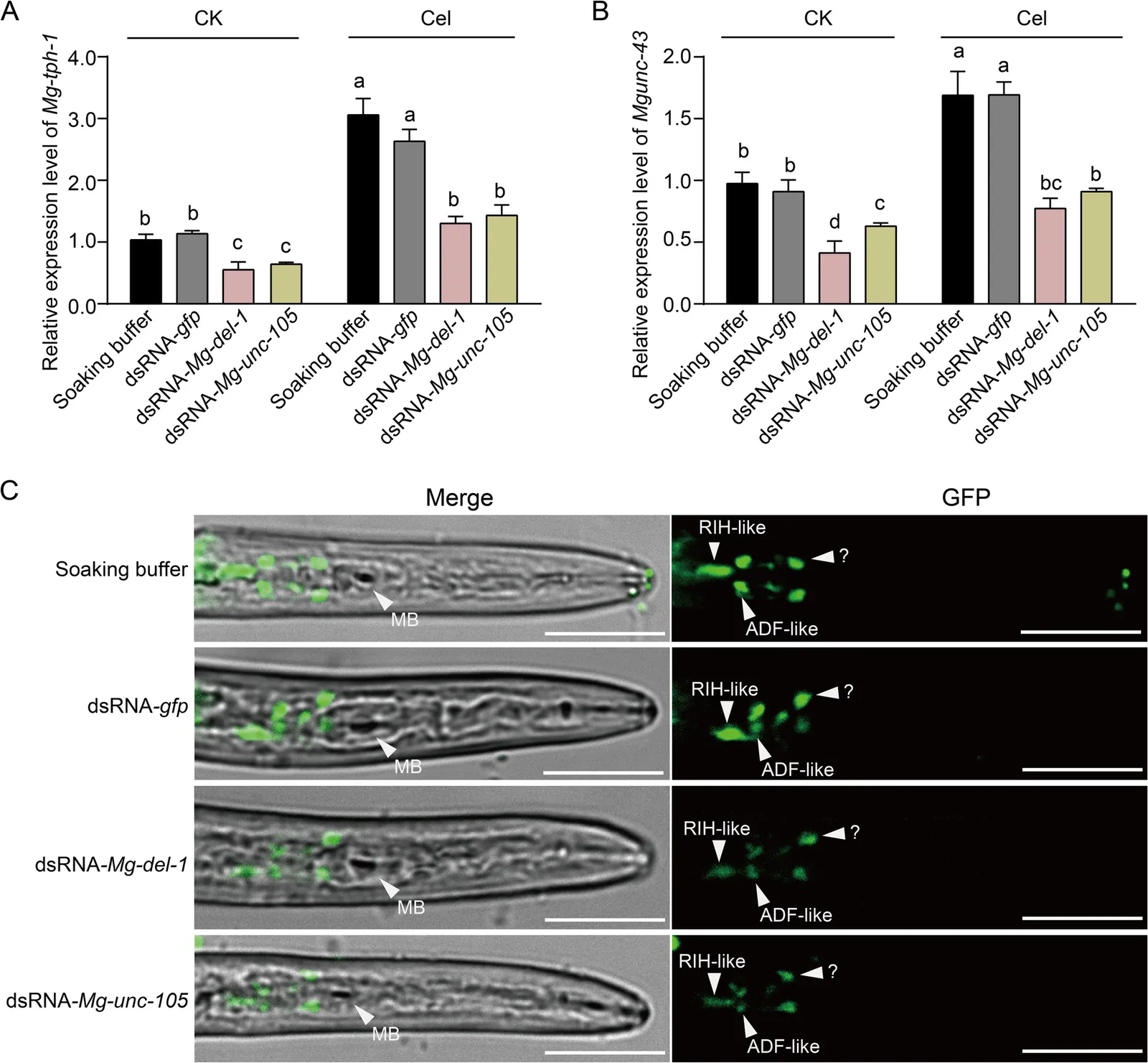
Effects of mechanosensory gene silencing on serotonergic pathway gene expression and serotonin (5‑HT) immunoreactivity in *M. graminicola* J2. Relative expression levels of the *Mg-tph-1* (tryptophan hydroxylase) (**A**) and *Mg-unc-43* (CaMKII homolog) (**B**) under different conditions. CK, sterile water control; Cell, treatment with 20 µg/mL cellulose. **C** Immunohistochemical localization of serotonin in nematodes after treatment with 20 µg/mL cellulose for 1 h. Arrowheads indicate serotonergic neurons (ADF-like and RIH-like); arrowheads with question marks denote structures of uncertain anatomical identity. Arrowheads indicate the median bulb (MB). Bar = 20 μm. Data are presented as mean ± standard deviations (SDs) from three independent experiments. Different lowercase letters above the bars indicate statistically significant differences among treatments (one‑way ANOVA followed by Duncan’s multiple range test, *p* < 0.05)

Consistent with our previous findings [29] that reported RNAi-mediated silencing of the calcium/calmodulin‑dependent kinase II (CaMKII) homolog *Mg-unc-43* led to significant downregulation of *Mg-tph-1* expression and loss of 5-HT immunoreactivity, this study found that silencing *Mg-del-1* or *Mg-unc-105* also reduced the transcript levels of *Mg-unc-43* and *Mg-tph-1*, accompanied by a marked reduction of 5-HT immunostaining signals (Fig. 5A–C). Notably, the neuronal distribution of 5-HT after one hour of cellulose treatment (Fig. 5C) mirrored that following one hour of RBCs treatment (Supplementary Fig. 3A).

## Discussion

The transition from host location to successful invasion is a critical yet poorly understood stage in the life cycle of PPNs. While chemical cues guide nematodes toward the root tip, their immediate behavioral response upon contact—characterized by rhythmic stylet thrusting—must be precisely regulated to penetrate plant cell walls. This study elucidates a previously uncharacterized mechanosensory pathway in *M. graminicola*, in which DEG/ENaC channels mediate the perception of mechanical cues from rice RBCs, primarily cellulose. Activation of these channels induces serotonin biosynthesis, thereby driving stylet thrusting and facilitates host invasion.

### RBCs as critical mechanical signals for pre-invasion behavior

RBCs and their secreted mucilage form a protective network at the root tip, serving as the plant’s first line of defense against soil-borne microbial pathogens. Originating from the root cap meristem, these specialized cells detach to surround the root tip as a discrete layer [30, 32]. Upon detachment, RBCs undergo significant physiological changes, decreasing root-soil friction and shielding the root apex from mechanical damage [22]. Beyond these structural roles, emerging evidence indicates that RBCs actively influence the behavior of PPNs, though these effects are highly host-dependent. For instance, maize RBCs enhance the motility of *M. incognita* on contact without strong chemotactic attraction [7], whereas pea RBCs attract the same nematode species and subsequently induce a quiescent state—a response absent in snap bean [8]. This diversity of interactions suggests that RBCs may act as physical modifiers, behavioral traps, or inert surfaces depending on host plant.

Our findings extend this paradigm by revealing that rice RBCs trigger stereotyped pre‑invasion behavior in *M. graminicola*—a response distinct from both the quiescent state induced in pea and the motility enhancement observed in maize. The robust induction of stylet thrusting by RBCs in our study contrasts with the quiescence induced in *M. incognita* by pea border cells [8], suggesting that different plant hosts have evolved distinct strategies to modulate nematode invasion behavior. Furthermore, while pea RBCs attract *M. incognita* and induce quiescence, and maize border cells enhance motility, our rice system showed only weak chemotactic attraction to RBCs at the highest density tested (CI = 0.15) (Supplementary Fig. 3B). This functional divergence implies that the signaling properties of RBCs are species-specific and that *M. graminicola* has adapted to interpret rice RBCs as an invasion-promoting cue rather than a deterrent or quiescence signal. Given that RBCs activate a serotonergic pathway to drive stylet activity in *M. graminicola* and considering the known role of serotonin in suppressing somatic locomotion in nematodes [20], we speculate that the quiescent state induced in *M. incognita* by pea border cells may similarly be mediated by serotonin, albeit through neuronal circuits that inhibit rather than promote stylet activity.

The RBC densities used in our in vitro assays (3 × 10¹ to 3 × 10⁵ cells/mL) span the physiological range reported for root tip microenvironments, where border cell numbers can reach several hundred to several thousand per root tip depending on species and developmental stage [23]. Thus, the observed RBC-induced responses are likely relevant to natural conditions at the root–soil interface. Pre-J2 rely on stored lipid reserves for survival prior to feeding site establishment, and failure to establish a functional feeding site ultimately results in death. While it is generally accepted that J2 begin ingesting nutrients only after a feeding site has been formed, our observation that RBCs induce Cy3 dye uptake suggests that stylet-associated ingestion—and potentially nutrient sampling—can occur prior to root penetration. Whether this early ingestion contributes to nematode survival or serves primarily as a behavioral readout of host recognition warrants further investigation.

### Cellulose: a dominant polysaccharide cue in mechanoperception

In addition to RBCs themselves, our identification of cellulose as the dominant polysaccharide cue eliciting stylet thrusting represents a mechanistic advance in understanding how nematodes recognize root surfaces. PPNs were the first animals shown to produce cell wall-modifying enzymes, including cellulases and pectinases, which they secrete through the stylet during migration and feeding site formation [33]. The cell wall, with its complex network of cellulose microfibrils, hemicelluloses, and pectin, constitutes the primary physical barrier nematodes must breach [33]. Traditionally, research has focused on chemosensory recognition of root exudates as the primary driver of host-seeking behavior [3, 4]. However, our data demonstrate that upon physical contact with the root tip, cellulose itself acts as a mechanical signal that triggers stylet thrusting.

This finding aligns with recent work in cucumber, where the CsBZR1-CsCEL1 module regulates susceptibility to *M. incognita* by mediating cellulose metabolism; suppression of CsCEL1 expression reduces β-1,4-endoglucanase activity and elevates root cellulose content, thereby enhancing nematode resistance [34]. While that study focused on cellulose content as a determinant of host susceptibility, our results go further by positioning cellulose as a direct mechanical cue perceived by the nematode. This insight reframes the plant–nematode interface: root cellulose is not merely an obstacle to be enzymatically degraded, but also an informational molecule that signals to the nematode that it has reached a suitable invasion site.

Notably, among the soluble sugars tested, glucose uniquely stimulated nematode dye uptake at a low concentration (2 µg/mL), whereas arabinogalactan promoted uptake in a concentration‑dependent manner (Fig. 2C–D). Interestingly, although monosaccharides and disaccharides triggered only weak stylet thrusting, they did induce dye uptake, indicating that stylet thrusting and ingestion are dissociable behaviors regulated by distinct sensory pathways. The induction of ingestion by soluble sugars may involve at least two non‑exclusive mechanisms. On one hand, in *C. elegans*, the insulin/IGF‑1 signaling (IIS) pathway regulates feeding behavior in response to nutrient availability [35], and exogenous glucose has been shown to affect IIS activity, leading to altered development, stress responses, and lifespan [36]. Although direct evidence is lacking for plant‑parasitic nematodes, it is plausible that soluble sugars such as glucose and arabinogalactan in the rhizosphere are perceived as nutritional cues and influence the ingestion behavior of *M. graminicola* through an insulin‑like signaling pathway. On the other hand, the observed ingestion response is also consistent with the established role of SWEET (Sugars Will Eventually be Exported Transporters) family proteins in sugar allocation during plant–nematode interactions [37]. In plants, SWEETs facilitate the efflux of sugars across plasma membranes and are critical for phloem loading and sugar distribution [38]. Upon nematode infection, multiple host SWEET genes are upregulated, and SWEET‑mediated sugar transport contributes to carbohydrate allocation to nematode‑induced feeding sites such as giant cells and syncytia [37, 39]. Moreover, recent functional characterization of *SWEET* genes in *M. incognita* revealed that specific *Mi‑SWEETs* support nutrient uptake during feeding, while others facilitate mobility and root invasion in motile juveniles [40]. These findings indicate that SWEET transporters play important roles in sugar acquisition at feeding sites during the parasitic stage—a process distinct from the pre‑invasion mechanosensory pathway that triggers stylet thrusting upon root contact.

Among the polysaccharides we tested, cellulose induced the strongest stylet thrusting response (80.7% of J2 at 20 µg/mL), while arabinogalactan also stimulated high thrusting frequency (45.3 thrusts/min at 20 µg/mL) (Fig. 2). Notably, arabinogalactan is a water‑soluble proteoglycan that may be rapidly diluted or washed away in the rhizosphere, whereas cellulose fibrils are physically anchored in the root cell wall, providing a stable and persistent mechanical stimulus. This distinction suggests that insoluble polysaccharides may serve as more reliable contact-dependent cues for initiating invasion behaviors. The aggregation phenotype induced by cellulose (Supplementary Fig. 5B) further supports this interpretation: once nematodes contact cellulose-rich root surfaces, they tend to cluster, potentially coordinating invasive stylet activity at invasion sites.

The sugar concentrations used in our study (2 and 20 µg/mL) fall within the range of soluble sugars reported in root exudates (1–500 µg·mL⁻¹) [41], and root tips are known to be hotspots of carbon secretion. Furthermore, root border cells have been shown to take up glucose across a concentration range of 0.2–20 mM [42], suggesting that root tip cells are associated with sugar-rich microenvironments. While direct in vivo equivalence is difficult to establish, the tested concentrations are physiologically plausible and may be encounterable by nematodes at the root–soil interface.

### Functional conservation and specialization of DEG/ENaC channels in parasitism

The identification of two DEG/ENaC-type mechanosensory channel genes, *Mg-del-1* and *Mg-unc-105*, establishes a molecular link between mechanical perception and behavioral output in *M. graminicola*. In the model organism *C. elegans*, the DEG/ENaC family has been extensively characterized as a major mechanotransduction pathway [15, 16, 43]. Different DEG/ENaC subunits serve distinct sensory functions: MEC‑4 and MEC‑10 form the core gentle touch transduction channel in sensory neurons [15, 16]; and DEGT‑1 functions as a proprioceptor monitoring pharyngeal movement to regulate feeding rate [17, 19].

Phylogenetic analysis placed Mg‑DEL‑1 and Mg‑UNC‑105 closely related to these *C. elegans* touch receptors (Supplementary Fig. 6), with Mg‑DEL‑1 sharing 50.79% similarity with *C. elegans* DEL‑1 and Mg‑UNC‑105 showing 44.57% similarity with *C. elegans* UNC‑105. This degree of conservation, coupled with the functional effects of RNAi silencing, suggests that these channels have been co‑opted during the evolution of plant parasitism to detect plant-derived mechanical cues rather than merely serving general tactile functions. The elevated expression of both genes in par-J2 compared to other stages suggests their involvement in feeding site establishment and the early parasitic process, rather than merely in pre-invasion host recognition. Their reduced expression in J3/J4 stages coincides with the transition to a sedentary lifestyle, a process associated with neuromuscular remodeling and muscle atrophy in other sedentary plant-parasitic nematodes such as *H. glycines* and *M. incognita* [44]. Moreover, the distinct expression patterns of the two genes indicate potential functional divergence. The par-J2‑biased expression of *Mg-del-1* implies a more specialized role during the parasitic phase, whereas the sustained expression of *Mg-unc-105* in both pre-J2 and par-J2 suggests broader functions spanning both pre‑ and post‑invasion stages. Notably, in situ hybridization revealed widespread expression of both genes, with particularly strong signals in the intestinal region and a distinct *Mg‑del‑1* signal in the median bulb (Fig. 3B), it should be noted that this technique may not have sufficient resolution to definitively exclude or confirm their expression in specific tissues or neurons. While the intestinal expression was unexpected for mechanosensory channels, it is worth noting that in *C. elegans*, some DEG/ENaC channels are expressed in non‑neuronal tissues and contribute to diverse physiological functions [18, 19, 43]. The median bulb localization of *Mg‑del‑1* is particularly intriguing, given that this structure houses the metacorpus pump involved in feeding—suggesting a potential role in monitoring feeding‑related mechanical forces.

Functional silencing of either *Mg‑del‑1* or *Mg‑unc‑105* using RNAi resulted in significant reductions in nematode motility, stylet thrusting, and feeding activity (Fig. 4). These phenotypes are reminiscent of touch‑insensitive *C. elegans* mutants lacking DEG/ENaC channel subunits [43]. However, an important distinction emerges: whereas *C. elegans* relies on these channels for sensing its own movements (proprioception) or external tactile stimuli, *M. graminicola* appears to have adapted these same molecular components to perceive plant‑derived cellulose, linking mechanical detection directly to parasitic behaviors. The transcript levels of *Mg-del-1* and *Mg-unc-105* were not induced by RBCs or cellulose exposure (Fig. 3A), suggesting that these channels are constitutively expressed and may be further post‑translationally regulated upon mechanical stimulation. This is consistent with the known behavior of DEG/ENaC channels, which are gated directly by membrane tension and extracellular mechanical forces, with channel opening triggering downstream signaling without requiring transcriptional upregulation [14, 45].

### Serotonin as a downstream effector of mechanosensory signaling

Our finding that silencing *Mg‑del‑1* or *Mg‑unc‑105* downregulates the serotonergic pathway genes *Mg‑unc‑43* and *Mg‑tph‑1* and reduces 5‑HT immunoreactivity (Fig. 5) provides a mechanistic link between mechanoperception and neurochemical modulation. The CaMKII homolog Mg‑UNC‑43 likely transduces the calcium signal initiated by DEG/ENaC channel activation. In both *C. elegans* and mammals, CaMKII plays a central role in translating calcium influx into prolonged cellular responses, including the regulation of neurotransmitter synthesis [46]. Our data suggest a signaling cascade in which Mg‑DEL‑1 and Mg‑UNC‑105, upon sensing mechanical forces from cellulose, permit calcium influx that activates Mg‑UNC‑43, which in turn upregulates the tryptophan hydroxylase gene *Mg‑tph‑1* (the rate‑limiting enzyme in serotonin biosynthesis). The resultant elevation in 5‑HT then acts on serotonergic receptors to drive stylet thrusting.

This model is supported by the observed activation and reconfiguration of serotonergic neurons following RBCs or cellulose exposure (Fig. 5C; Supplementary Fig. 3A). The dynamic emergence of specific serotonergic neurons—ADF‑like, RIH‑like, and later NSM‑like neurons—mirrors the pattern described in *M. graminicola* by Yang et al. (2023), where these neurons were proposed to be homologous to *C. elegans* ADF, RIH, and NSM serotonergic neurons. In *C. elegans*, ADF neurons regulate dauer formation and modulate chemotaxis, RIH neurons are involved in feeding and locomotion, and NSM neurons are mechanosensory neurons that release serotonin to stimulate pharyngeal pumping during feeding [47]. The recruitment of these homologous neuron types upon mechanical stimulation in *M. graminicola* suggests that the serotonergic control of feeding‑related behaviors is evolutionarily conserved but has been repurposed in plant‑parasitic nematodes to coordinate stylet thrusting during host invasion.

This conclusion aligns with independent evidence from other plant‑parasitic nematode species. In *G. pallida*, pharmacological disruption of serotonin signaling using reserpine compromised the rhythmic stylet thrusting required for host penetration, demonstrating that serotonergic regulation of stylet activity is not unique to *M. graminicola* but may represent a conserved feature across diverse plant‑parasitic lineages [48]. Furthermore, a recent study by Xiao et al. (2026) demonstrated that silencing *Mg‑unc‑43* similarly reduced *Mg‑tph‑1* expression and impaired stylet thrusting, placing Mg‑UNC‑43 genetically upstream of *Mg‑tph‑1* in the same signaling pathway. Collectively, these findings establish a coherent neurobiological framework: mechanical detection via DEG/ENaC channels feeds into a conserved CaMKII to serotonin signaling axis that ultimately drives the motor output required for root invasion.

**Fig. 6:**
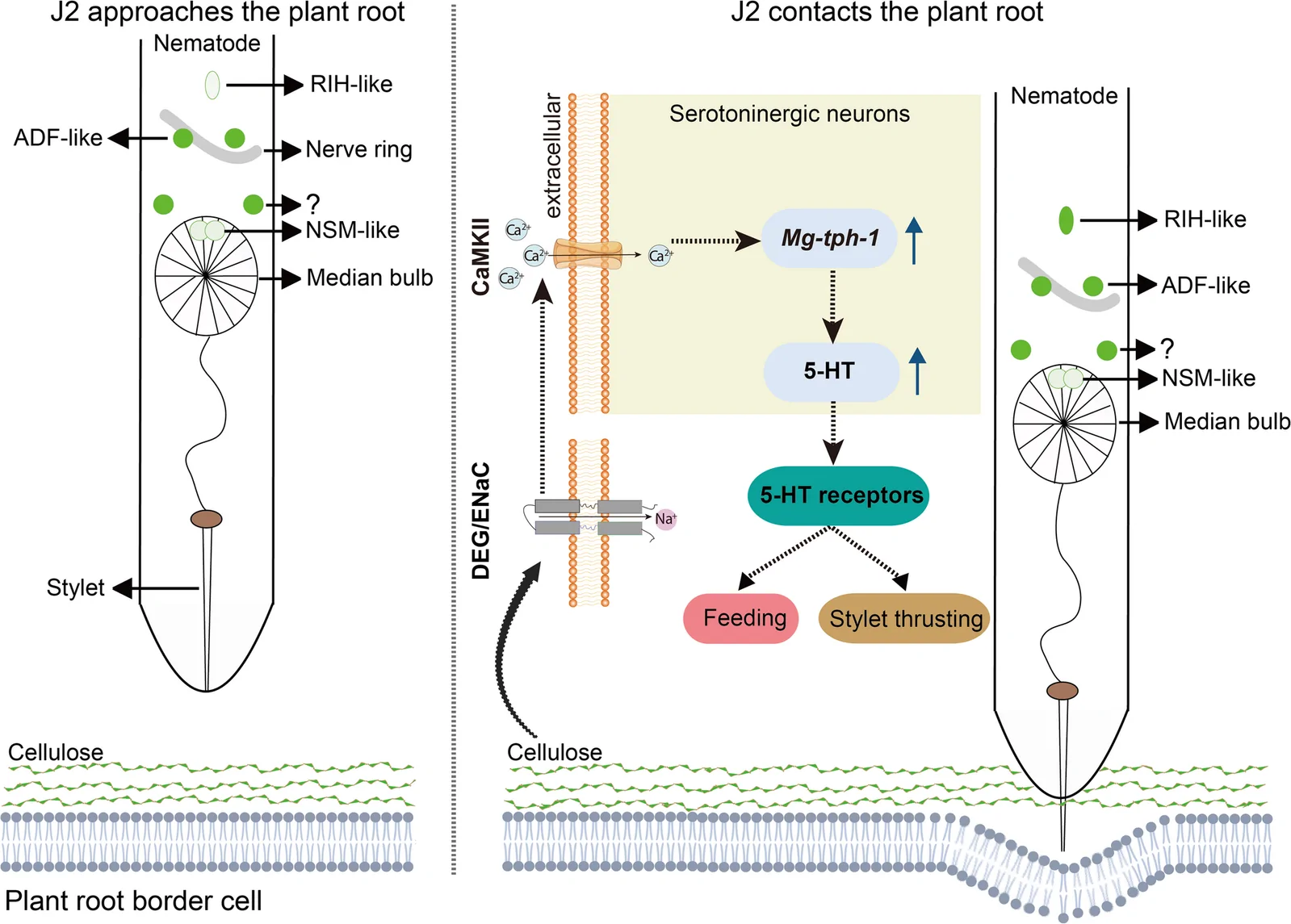
Proposed model for *M. graminicola* J2 perception and response to rice root tip signals. The infective second-stage juvenile (J2) is attracted to the rice root tip and makes contact with root border cells. Mechanical cues (cellulose) from the cell wall are perceived by putative mechanosensory channels (DEG/ENaC). This signal may be transduced via calcium influx through channels such as CaMKII homolog Mg-UNC-43. This signal is integrated within the serotonergic neurons (ADF-like) and the upregulation of *Mg-tph-1*, thereby elevating serotonin (5-HT) levels. The resulting increase in 5-HT activates downstream serotonergic receptors, which in turn promotes feeding and stylet thrusting

Based on the integrated experimental evidence, we propose a working model in which the infective J2 approaches the rice root tip and contacts RBCs (Fig. 6). Insoluble cellulose fibrils in the cell wall provide a stable mechanical cue, and the resulting mechanical force is transduced Mg‑DEL‑1 and Mg‑UNC‑105 mechanosensory channels. Channel activation permits calcium influx, which is transduced via the CaMKII homolog Mg‑UNC‑43, leading to upregulation of *Mg‑tph‑1* and enhanced serotonin biosynthesis. The newly synthesized 5‑HT is released from specific serotonergic neurons (ADF‑like, RIH‑like) and acts on downstream receptors to stimulate the motor circuitry controlling rhythmic stylet thrusting, thereby facilitating root penetration and feeding site establishment. The fact that silencing these channels phenocopies the effects of silencing *Mg‑tph‑1* [20] or *Mg‑unc‑43* [29] strengthens the genetic epistasis within this pathway. Moreover, the high sequence conservation of Mg‑DEL‑1 and Mg‑UNC‑105 with homologs in other root‑knot nematode species (Supplementary Fig. 7–8) raises the possibility that this mechanosensory circuit is conserved among *Meloidogyne* species and perhaps across other plant‑parasitic nematode genera. Given the severe economic losses caused by PPNs worldwide each year [1], targeting mechanosensory channels involved in invasion behavior opens new avenues for nematode control. Small‑molecule inhibitors targeting DEG/ENaC channels or components of the downstream serotonergic pathway could potentially disrupt stylet activity at the earliest stage of parasitism, providing a novel strategy for reducing nematode infection.

Beyond the immediate behavioral responses observed in J2, an intriguing question is whether variation in carbohydrate availability influences the male-to-female ratio during nematode development. Recent evidence suggests that sugar availability plays a role in sex determination in plant-parasitic nematodes. In *M. incognita*, sugar-limiting conditions promote male development, with a strong correlation observed between reduced giant cell development and male differentiation [49]. Similarly, in the cyst nematode *G. pallida*, manipulating host sucrose availability can steer sex determination, with high sucrose promoting female development [50]. Although these two species differ in their feeding-site structures (giant cells vs. syncytia) and parasitic strategies, the sex-determination mechanisms identified in both studies operate at the post-establishment level, relying on nutrient availability within established feeding sites rather than on pre-invasion carbohydrate perception. Thus, although their experimental systems do not directly align with our pre-penetration sugar exposure assays, these findings nevertheless raise the intriguing possibility that carbohydrate signals encountered prior to root entry could have lasting consequences for developmental fate. While it remains unclear whether simple versus complex sugars differentially affect this process in *M. graminicola*, these findings raise the possibility that carbohydrate perception during early parasitism may have long-term consequences for population structure. Future studies employing suitable experimental systems could help dissect the mechanistic link between carbohydrate signals and developmental fate.

While our study establishes a functional mechanosensory pathway in *M. graminicola*, several important questions remain. First, the precise subcellular localization of Mg‑DEL‑1 and Mg‑UNC‑105—whether they are expressed in the sensory neurons that directly contact the root surface, and whether they co‑localize in the same cellular compartments—requires high‑resolution immunolocalization studies. Second, although our genetic epistasis data place Mg‑DEL‑1 and Mg‑UNC‑105 upstream of *Mg‑unc‑43* and *Mg‑tph‑1*, direct protein‑protein interactions among these components have not been demonstrated. Yeast two‑hybrid or co‑immunoprecipitation experiments could clarify whether these channels form a physical complex with Mg‑UNC‑43 or whether their relationship is mediated indirectly through calcium signaling. Third, the relative contributions of *Mg‑del‑1* and *Mg‑unc‑105* to different aspects of mechanosensation (stylet thrusting vs. locomotion vs. feeding) warrant further dissection, potentially through tissue‑specific RNAi or knockout approaches. Fourth, it remains to be determined whether other RBC‑derived polysaccharides, such as arabinogalactan, act through the same DEG/ENaC channels or through distinct receptors. Fifth, and most importantly for translational applications, screening for compounds that selectively modulate DEG/ENaC channel activity without affecting beneficial soil nematodes could yield specific inhibitors that disrupt mechanosensory‑driven parasitism. Such an approach would complement existing nematicides that target chemosensation or metabolic pathways, offering a mechanism‑based strategy for sustainable nematode management. Finally, while our genetic and behavioral data support a model (Fig. 6) in which DEG/ENaC-mediated mechanosensation leads to calcium influx and downstream serotonin biosynthesis, direct evidence of in vivo calcium signaling upon cellulose perception is currently lacking. In *C. elegans*, calcium imaging using genetically encoded indicators such as GCaMP has been successfully employed to monitor real-time calcium dynamics in mechanosensory neurons in response to mechanical stimuli [51]. Adapting similar approaches in *M. graminicola* would enable direct visualization of calcium transients upon cellulose or RBCs contact. Pharmacological interventions using calcium chelators or channel blockers could also provide complementary evidence. Nonetheless, our combined genetic, behavioral, and immunocytochemical data establish a coherent working model that provides a framework for future investigations at the cellular and molecular levels.

## Conclusions

In conclusion, this study uncovers a mechanosensory channel‑mediated signaling axis that is crucial for the early parasitism of *M. graminicola*. It advances our understanding of host recognition by plant‑parasitic nematodes beyond chemotaxis, introducing physical cue perception as a key determinant of invasion behavior. Our working model positions DEG/ENaC channels as critical upstream sensors, offering promising molecular targets for disrupting the very first step of nematode invasion. Future studies should investigate how *M. graminicola* perceives mechanical signals from internal root cells and utilizes carbohydrates within the root tissue, as these processes may differ fundamentally from the pre‑invasion responses characterized in the present study.

## Supplementary Information

Supplementary Material 1.

Supplementary Material 2: Supplementary Table 1. Primers used in this study. Supplementary Figure 1. Rice root border cells induce M. graminicola stylet thrusting. (A) Relative expression of Mg-tph-1 gene. (B) Frequency of stylet thrusts. CK, water control; 25 mM serotonin (5-HT), positive control. The experiment was repeated three times. Data are presented as the mean ± standard deviation (calculated from the pooled data of three independent replicates). Different letters indicate significant differences determined by Duncan’s multiple range test. Supplementary Figure 2. Statistics on the number and survival rate of root tip border cells. (A) Statistical analysis of root border cell numbers at different root lengths. (B) Statistical analysis of root border cell survival rates at different root lengths. The rice varieties included Nongken58 (NK58), Nipponbare (NPB), Huanghuazhan (HHZ), IR64, Zhonghua11 (ZH11), and Huaidao5 (HD5). Bar = 200 µm. The experiment was repeated twice, and the data are presented as the mean ± standard deviations (pooled from two independent experiments). The different letters indicate significant differences using Duncan's multiple range test (*p* < 0.05). Supplementary Figure 3. Chemotaxis of M. graminicola towards rice root border cells and distribution of serotonergic neurons. (A) Distribution of serotonergic neurons in M. graminicola after 1-hour and 2-hour contact with rice root border cells. MB, median bulb. Bar = 20 µm. (B) Chemotaxis analysis of M. graminicola towards different concentrations of root border cells. The experiment was repeated three times. Data are presented as the mean ± standard deviation (calculated from the pooled data of three independent replicates). Different letters indicate significant differences determined by Duncan’s multiple range test. Supplementary Figure 4. Morphology of rice root tip and monosaccharide composition of root border cells. (A) Morphology of a rice root tip section. The section was stained with toluidine blue. Bar = 100 μm. (B) Monosaccharide composition profiles of root border cells from NPB and IR64 varieties. Gal, galactose; Ara, arabinose; Xyl, xylose; Glc, glucose; Fuc, fucose; Man, mannose; Rha, rhamnose; Glu-UA, glucuronic acid; Gal-UA: Galacturonic acid. Data are presented as means ± SD from three independent biological replicates. Supplementary Figure 5. Analysis of nematode chemotaxis and aggregation in response to different sugars. (A) Chemotaxis assay of M. graminicola toward various sugars. (B) Induction of aggregation behavior in M. graminicola by different sugars. The tested sugars include: Xyl (xylose), Ara (arabinose), Gal (galactose), Glc (glucose), Suc (sucrose), Mal (maltose), Cel (cellulose), AG (arabinogalactan), and Pec (pectin). Bar = 500 µm. Data are presented as the mean ± SD (pooled from two independent experiments). Different letters indicate significant differences among treatments using Duncan's multiple range test (*P* < 0.05). Supplementary Figure 6. Phylogenetic tree of predicted Mg-DEL-1 and Mg-UNC-105 proteins. Complete amino acid sequences were aligned using ClustalW. The phylogenetic tree was constructed via the Maximum Likelihood method with 1000 bootstrap replicates in MEGA7.0. The Mg-DEL-1 and Mg-UNC-105 of M. graminicola were highlighted in red. Supplementary Figure 7. Multiple sequence alignment of Mg-DEL-1 with homologous proteins. Sequence alignment of Mg-DEL-1 (M. graminicola), DEL-1 (C. elegans, NP_001355359.1) and M. enterolobii (CAK5091297.1) performed with Clustal X v2.1. Residues highlighted in yellow represent the conserved Amiloride-sensitive sodium channel domain. Supplementary Figure 8. Multiple sequence alignment of Mg-UNC-105 with homologous proteins. Sequence alignment of Mg-UNC-105 (M. graminicola), UNC-105 (C. elegans, NP_001254139.1) and M. hapla (KAL7071408.1) performed with Clustal X v2.1. Residues highlighted in yellow represent the conserved Amiloride-sensitive sodium channel domain.

## Data Availability

All data generated or analyzed during this study are included in this published article and its supplementary information files.

## Abbreviations

5-HT: Serotonin (5‑hydroxytryptamine)
CaMKII: Calcium/calmodulin‑dependent kinase II
DEG/ENaC: Degenerin/Epithelial Sodium Channel
J2: Second‑stage juvenile
pre‑J2: Preparasitic second‑stage juvenile
RBCs: Root border cells
RNAi: RNA interference

## References

[1] Jones J, Haegeman A, Danchin E, Gaur HS, Helder J, Jones M, Kikuchi T, Manzanilla Lopez RH, Palomares-Rius J, Wesemael W et al. Top 10 plant-parasitic nematodes in molecular plant pathology. Mol Plant Pathol. 2013;14(9):946–61. 10.1111/mpp.12057 PMC6638764 23809086

[2] Rusinque L, Maleita C, Abrantes I, Palomares-Rius JE, Inácio ML. *Meloidogyne graminicola*-A Threat to Rice Production: Review Update on Distribution, Biology, Identification, and Management. Biology. 2021;10(11):1163. 10.3390/biology10111163 PMC8614973 34827156

[3] Bais H, Weir T, Perry L, Gilroy S, Vivanco J. The Role of Root Exudatesin Rhizosphere Interactions with Plants and Other Organisms. Annu Rev Plant Biol. 2006;57:233–66. 10.1146/annurev.arplant.57.032905.105159 16669762

[4] Reynolds AM, Dutta TK, Curtis RH, Powers SJ, Gaur HS, Kerry BR. Chemotaxis can take plant-parasitic nematodes to the source of a chemo-attractant via the shortest possible routes. J R Soc Interface. 2011;8(57):568–77. 10.1098/rsif.2010.0417 PMC3061123 20880854

[5] Omelyanchuk NA, Cherenko VA, Zemlyanskaya EV. Root cap border cells as regulators of rhizosphere microbiota. Vavilovskii zhurnal genetiki i selektsii. 2024;28(8):918–26. 10.18699/vjgb-24-99 PMC11811504 39944807

[6] Driouich A, Gueye M-LF, Vicré M, Moore JP. The root extracellular trap; a complex and dynamic biomatrix network essential for plant protection. Curr Opin Plant Biol. 2024;82:102656. 10.1016/j.pbi.2024.102656 39536647

[7] Rodger S, Bengough AG, Griffiths BS, Stubbs V, Young IM. Does the Presence of Detached Root Border Cells of Zea mays Alter the Activity of the Pathogenic Nematode Meloidogyne incognita? Phytopathology. 2003;93(9):1111–4. 10.1094/PHYTO.2003.93.9.1111 18944094

[8] Zhao X, Schmitt M, Hawes MC. Species-dependent effects of border cell and root tip exudates on nematode behavior. Phytopathology. 2000;90(11):1239–45. 10.1094/PHYTO.2000.90.11.1239 18944426

[9] Schafer WR. Mechanosensory molecules and circuits in *C. elegans*. Pflug Arch: Eur J Physiol. 2015;467(1):39–48. 10.1007/s00424-014-1574-3 PMC4281349 25053538

[10] Patkunarajah A, Stear JH, Moroni M, Schroeter L, Blaszkiewicz J, Tearle JL, Cox CD, Fürst C, Sánchez-Carranza O, Ocaña Fernández M et al. TMEM87a/Elkin1, a component of a novel mechanoelectrical transduction pathway, modulates melanoma adhesion and migration. eLife. 2020;9:e53308. 10.7554/eLife.53308 PMC7173973 32228863

[11] Heppelmann B, McDougall JJ. Inhibitory effect of amiloride and gadolinium on fine afferent nerves in the rat knee: evidence of mechanogated ion channels in joints. Exp Brain Res. 2005;167(1):114–8. 10.1007/s00221-005-0040-z 16025293

[12] Li W, Kang L, Piggott BJ, Feng Z, Xu XZ. The neural circuits and sensory channels mediating harsh touch sensation in *Caenorhabditis elegans*. Nat Commun. 2011;2:315. 10.1038/ncomms1308 PMC3098610 21587232

[13] Bazopoulou D, Voglis G, Tavernarakis N. The Role of DEG/ENaC Ion Channels in Sensory Mechanotransduction. In: *Molecular Sensors for Cardiovascular Homeostasis.* Edited by Wang DH. Boston, MA: Springer US; 2007: 3–31.

[14] Chen Y, Bharill S, Isacoff EY, Chalfie M. Subunit composition of a DEG/ENaC mechanosensory channel of *Caenorhabditis elegans*. Proc Natl Acad Sci USA. 2015;112(37):11690–5. 10.1073/pnas.1515968112 PMC4577206 26324944

[15] Bianchi L. Mechanotransduction: touch and feel at the molecular level as modeled in *Caenorhabditis elegans*. Mol Neurobiol. 2007;36(3):254–71. 10.1007/s12035-007-8009-5 17955200

[16] O’Hagan R, Chalfie M, Goodman MB. The MEC-4 DEG/ENaC channel of *Caenorhabditis elegans* touch receptor neurons transduces mechanical signals. Nat Neurosci. 2005;8(1):43–50. 10.1038/nn1362 15580270

[17] Tao L, Porto D, Li Z, Fechner S, Lee SA, Goodman MB, Xu XZS, Lu H, Shen K. Parallel Processing of Two Mechanosensory Modalities by a Single Neuron in *C. elegans*. Dev Cell. 2019;51(5):617–e631613. 10.1016/j.devcel.2019.10.008 PMC8635448 31735664

[18] Bayer EA, Mango SE, Hobert O, Schier AF. The DEG/ENaC channel DEGT-1 is a proprioceptor of *C. elegans* foregut movement. Curr biology: CB. 2025;35(21):5332–e53435335. 10.1016/j.cub.2025.09.065 41135517

[19] Bayer EA, Mango SE, Hobert O, Schier AF. The mechanosensory DEG/ENaC channel DEGT-1 is a proprioceptor of *C. elegan* foregut movement. *bioRxiv* 2025:2025.2001.2001.631014. 10.1016/j.cub.2025.09.065 41135517

[20] Yang Z, Zhang Z, Long X, Shi X, Wang D, Peng D, Ye S, Ding Z. Clarifying the functional role of serotonin in *Meloidogyne graminicola* host plant parasitism by immunolocalization and RNA interference. Phytopathology^®^. 2023. 10.1094/PHYTO-08-23-0290-R 38148161

[21] Wang D, Shi X, Long X, Zhang Z, Ye S, Ding Z, Yang Z. Effect of RNA interference targeting chemosensory genes on chemotactic host-seeking in *Meloidogyne graminicola*. Eur J Plant Pathol. 2024. 10.1007/s10658-024-02818-z.

[22] Driouich A, Follet-Gueye ML, Vicré-Gibouin M, Hawes M. Root border cells and secretions as critical elements in plant host defense. Curr Opin Plant Biol. 2013;16(4):489–95. 10.1016/j.pbi.2013.06.010 23856080

[23] Hawes M, Allen C, Turgeon BG, Curlango-Rivera G, Minh Tran T, Huskey DA, Xiong Z. Root Border Cells and Their Role in Plant Defense. Annu Rev Phytopathol. 2016;54:143–61. 10.1146/annurev-phyto-080615-100140 27215971

[24] Haegeman A, Bauters L, Kyndt T, Rahman MM, Gheysen G. Identification of candidate effector genes in the transcriptome of the rice root knot nematode *Meloidogyne graminicola*. Mol Plant Pathol. 2013;14(4):379–90. 10.1111/mpp.12014 PMC6638898 23279209

[25] Livak KJ, Schmittgen TD. Analysis of relative gene expression data using real-time quantitative PCR and the 2(-Delta Delta C(T)) Method. Methods (San Diego Calif). 2001;25(4):402–8. 10.1006/meth.2001.1262 11846609

[26] Rosso M-N, Dubrana M-P, Cimbolini N, Jaubert-Possamai S, Abad P. Application of RNA Interference to Root-Knot Nematode Genes Encoding Esophageal Gland Proteins. Mol plant-microbe interactions: MPMI. 2005;18:615–20. 10.1094/MPMI-18-0615 16042006

[27] Yang Z, Xiao Q, Wang Y, Zhang Y, Ye S, Sun P, Zhang W, Deng H, Liu S, Ding Z. Integrated transcriptome and BSA-seq analysis identifies a novel QTL for *Meloidogyne graminicola* resistance in rice HuaHang31. Theor Appl Genet. 2025;138(9):208. 10.1007/s00122-025-04999-5 40788376

[28] Kimber MJ, McKinney S, McMaster S, Day TA, Fleming CC, Maule AG. flp gene disruption in a parasitic nematode reveals motor dysfunction and unusual neuronal sensitivity to RNA interference. FASEB journal: official publication Federation Am Soc Experimental Biology. 2007;21(4):1233–43. 10.1096/fj.06-7343com 17200420

[29] Xiao W, Wang Y, Long X, Ye S, Ding Z, Yang Z. The CaMKII homologue Mg-unc-43 mediates parasitism in *Meloidogyne graminicola* by regulating locomotion and stylet thrusting. Nematology. 2026;28(2):117–29.

[30] Feldman LJ. The development and dynamics of the root apical meristem. Am J Bot. 1984;71(9):1308–14. 11540826

[31] Chaboud A, Rougier M. Identification and Localization of Sugar Components of Rice (*Oryza sativa* L.) Root Cap Mucilage. J Plant Physiol. 1984;116(4):323–30. 10.1016/S0176-1617(84)80111-X 23195302

[32] Driouich A, Gaudry A, Pawlak B, Moore JP. Root cap-derived cells and mucilage: a protective network at the root tip. Protoplasma. 2021;258(6):1179–85. 10.1007/s00709-021-01660-y 34196784

[33] Wieczorek K. Chapter Three - Cell Wall Alterations in Nematode-Infected Roots. In: *Advances in Botanical Research.* Edited by Escobar C, Fenoll C, vol. 73: Academic Press; 2015: 61–90.

[34] Ji T, Liang M, Li S, Wang X, Cui L, Bu Y, Gao L, Ma S, Tian Y. CsBZR1-CsCEL1 module regulates the susceptibility of cucumber to Meloidogyne incognita by mediating cellulose metabolism. Plant journal: cell Mol biology. 2025;121(6):e70094. 10.1111/tpj.70094 40121570

[35] Murphy CT, Hu PJ. Insulin/insulin-like growth factor signaling in *C. elegans*. WormBook: online Rev C elegans biology 2013:1–43. 10.1895/wormbook.1.164.1 PMC4780952 24395814

[36] Schulz T, Zarse K, Voigt A, Urban N, Birringer M, Ristow M. Glucose Restriction Extends *Caenorhabditis elegans* Life Span by Inducing Mitochondrial Respiration and Increasing Oxidative Stress. Cell Metabol. 2007;6:280–93. 10.1016/j.cmet.2007.08.011 17908557

[37] Zhao D, You Y, Fan H, Zhu X, Wang Y, Duan Y, Xuan Y, Chen L. The Role of Sugar Transporter Genes during Early Infection by Root-Knot Nematodes. Int J Mol Sci. 2018;19(1):302. 10.3390/ijms19010302 PMC5796247 29351253

[38] Chen LQ, Hou BH, Lalonde S, Takanaga H, Hartung ML, Qu XQ, Guo WJ, Kim JG, Underwood W, Chaudhuri B, et al. Sugar transporters for intercellular exchange and nutrition of pathogens. Nature. 2010;468(7323):527–32. 10.1038/nature09606 PMC3000469 21107422

[39] Wang X, Wang Z, Tang X, Qin J, Zhou X, Gu L, Bian H, Sun L, Huang H, Yang R, et al. The SlDOF9-SlSWEET17 Module: a Switch for Controlling Sugar Distribution Between Nematode Induced Galls and Roots in Tomato. Adv Sci (Weinheim Baden-Wurttemberg Germany). 2025;12(28):e2501771. 10.1002/advs.202501771 PMC12302606 40349184

[40] Maxwell MWH, Rohilla B, Chippendale J, Bell CA. The stage-specific regulation and role of root-knot nematode SWEET genes. PLoS Pathog. 2026;22(5):e1014161. 10.1371/journal.ppat.1014161 PMC13148671 42090392

[41] Li X, Chu W, Dong J, Duan Z. An Improved High-performance Liquid Chromatographic Method for the Determination of Soluble Sugars in Root Exudates of Greenhouse Cucumber Grown under CO2 Enrichment. J Am Soc Hortic Sci. 2014;139:356–63.

[42] Stubbs VE, Standing D, Knox OG, Killham K, Bengough AG, Griffiths B. Root border cells take up and release glucose-C. Ann Botany. 2004;93(2):221–4. 10.1093/aob/mch019 PMC4241074 14726419

[43] Arnadóttir J, O’Hagan R, Chen Y, Goodman MB, Chalfie M. The DEG/ENaC protein MEC-10 regulates the transduction channel complex in *Caenorhabditis elegans* touch receptor neurons. J neuroscience: official J Soc Neurosci. 2011;31(35):12695–704. 10.1523/JNEUROSCI.4580-10.2011 PMC3172708 21880930

[44] Han Z, Thapa S, Reuter-Carlson U, Reed H, Gates M, Lambert KN, Schroeder NE. Immobility in the sedentary plant-parasitic nematode *H. glycines* is associated with remodeling of neuromuscular tissue. PLoS Pathog. 2018;14(8):e1007198. 10.1371/journal.ppat.1007198 PMC6095618 30114260

[45] Chatzigeorgiou M, Grundy L, Kindt KS, Lee WH, Driscoll M, Schafer WR. Spatial asymmetry in the mechanosensory phenotypes of the *C. elegans* DEG/ENaC gene mec-10. J Neurophysiol. 2010;104(6):3334–44. 10.1152/jn.00330.2010 PMC3007656 20881202

[46] Nicoll RA, Schulman H. Synaptic memory and CaMKII. Physiol Rev. 2023;103(4):2877–925. 10.1152/physrev.00034.2022 PMC10642921 37290118

[47] Song BM, Avery L. Serotonin activates overall feeding by activating two separate neural pathways in *Caenorhabditis elegans*. J neuroscience: official J Soc Neurosci. 2012;32(6):1920–31. 10.1523/JNEUROSCI.2064-11.2012 PMC3463504 22323705

[48] Crisford A, Calahorro F, Ludlow E, Marvin JMC, Hibbard JK, Lilley CJ, Kearn J, Keefe F, Johnson P, Harmer R, et al. Identification and characterisation of serotonin signalling in the potato cyst nematode Globodera pallida reveals new targets for crop protection. PLoS Pathog. 2020;16(10):e1008884–1008884. 10.1371/journal.ppat.1008884 PMC7556481 33007049

[49] Manabe K, Miyama A, Sawa S, Shinya R. Sugar availability influences sex determination in the root-knot nematode, *Meloidogyne incognita*, via changes in giant cell development. Nematology. 2025;27:895–904.

[50] Schaveling AS, van de Ruitenbeek SJS, Smant G, Sterken MG. Environmental sex determination in the cyst nematode *Globodera pallida* defaults to male development. BMC Biol. 2026. 10.1186/s12915-026-02671-y. PMC13632290 42399953

[51] Chatzigeorgiou M, Yoo S, Watson JD, et al. Specific roles for DEG/ENaC and TRP channels in touch and thermosensation in *C. elegans* nociceptors. Nat Neurosci. 2010;13(7):861–8. 10.1038/nn.2581. PMC2975101 20512132

