# Supplementary material for: Mechanosensation of rice root border cells activates serotonin-mediated pre-invasion behaviors in Meloidogyne graminicola via DEG/ENaC channels
Source: BMC Plant Biol. 2026 Jul 25;26:1812. doi: 10.1186/s12870-026-09616-1 (PMC13685373; doi:10.1186/s12870-026-09616-1)
Supplement: Supplementary file 1 [file 12870_2026_9616_MOESM2_ESM.docx]

**Supporting Information**

**Supplementary Table 1.** Primers used in this study.

| Primer | Sequence 5'- 3' | Purpose |
| --- | --- | --- |
| *Mg-del-1*-F | TCTGATCCAAGATATCCCCAAC | PCR amplification of *Mg-del-1* gene fragments encoding functional domains. |
| *Mg-del-1*-R | CAAGCTGGATTATGGTTAGGTGCA |  |
| *Mg-del-1*-2-F | AAGTAAAGAAAGTATTAGTAATACAAC |  |
| *Mg-del-1*-2-R | TTAAGCTTCATAAGGATTTTCA |  |
| SL1 | GGTTTAATTACCCAAGTTTGAG | Gene 5' flanking region PCR amplification. |
| 5’*Mg-unc-105*-R | CGAATAGTTGTAGCCTGT |  |
| 5’*Mg-del-1*-R | GTTGGGGATATCTTGGAT |  |
| 3′ RACE outer primer | TACCGTCGTTCCACTAGTGATTT | Gene 3' flanking region PCR amplification. |
| 3′ RACE inner primer | CGCGGATCCTCCACTAGTGATTTCACTATAGG |  |
| *Mg-unc-105*-outer | TTTGACACATTCGCTAAGGCAC |  |
| *Mg-unc-105*-inner | TTGCCCACAACGGAGGCTATTG |  |
| *Mg-del-1*-outer | TCTGATCCAAGATATCCCCAAC |  |
| *Mg-del-1*-inner | CAAGCTGGATTATGGTTAGGTGCA |  |
| *Mg-unc-105*-F BamHI | CGGGATCCTCCGACACTTCCTATTCC | For *in situ* localization |
| *Mg-unc-105*-R HindIII | CCCAAGCTTAACCCGTCTTTACGCTTG |  |
| *Mg-del-1-*F BamHI | CGGGATCCGAAATAAAATACCTGAAGAA |  |
| *Mg-del-1*-R EcoRI | GGAATTCTGAATAACAATATGAACACC |  |
| *Mg-Actin2*-F | ATGGCAACTGCCGCTTCTTCT | *M. graminicola* housekeeping gene (*ACT2*) used as qRT-PCR reference. |
| *Mg-Actin2*-R | AGATTCCGGACAACGGAAGCGT |  |
| q*Mg-unc-43*-F | GTTGTTCGTCGTTGTGTTCATAA | qRT-PCR for *Mg-unc-43* expression in *M. graminicola*. |
| q*Mg-unc-43*-R | ATCTTCAAAAAGTTCACCTCCAG |  |
| q*Mg-tph-1*-F | CTCTTTCAGGTGCTCCTTGGTTTCC | qRT-PCR for *Mg-tph-1* expression in *M. graminicola*. |
| q*Mg-tph-1*-R | GCATCAAGTCCTCCAGCTCCATAC |  |
| q*Mg-unc-105*-F | ATGGCGGAGGAACGGTTAGAC | qRT-PCR for *Mg-unc-105* expression in *M. graminicola*. |
| q*Mg-unc-105*-R | ATCGAGCGGCAGAATATGTAAGC |  |
| q*Mg-del-1*-F | TCGTCCAACACCTCCAATGC | qRT-PCR for *Mg-del-1* expression in *M. graminicola*. |
| q*Mg-del-1*-R | ACGTGTAGGTGTTGGTCCAC |  |
| dsRNA-*gfp*-F | TAATACGACTCACTATAGGGACCAACAAGATGAAGAGCAC | dsRNAs synthesis. |
| dsRNA*-gfp*-R | TAATACGACTCACTATAGGGTACATCGCTCTTTCTTCACCG |  |
| dsRNA-*Mg-del-1*-F | TAATACGACTCACTATAGGGAACGCAAATGATGTTTGT |  |
| dsRNA-*Mg-del-1*-R | TAATACGACTCACTATAGGGTTTCTGATGTTGGTGGAC |  |
| dsRNA-*Mg-unc-105*-F | TAATACGACTCACTATAGGGGTGGTGCCAGATAGGGTG |  |
| dsRNA-*Mg-unc-105*-R | TAATACGACTCACTATAGGGGCTGATTTGGGTGGGTTA |  |

**Supplementary Figure 1. Rice root border cells induce *Meloidogyne graminicola* stylet thrusting.** (A) Relative expression of *Mg-tph-1* gene. (B) Frequency of stylet thrusts. CK, water control; 25 mM serotonin (5-HT), positive control. The experiment was repeated three times. Data are presented as the mean ± standard deviation (calculated from the pooled data of three independent replicates). Different letters indicate significant differences determined by Duncan’s multiple range test.

**Supplementary Figure 2. Statistics on the number and survival rate of root tip border cells.** (A) Statistical analysis of root border cell numbers at different root lengths. (B) Statistical analysis of root border cell survival rates at different root lengths. The rice varieties included Nongken58 (NK58), Nipponbare (NPB), Huanghuazhan (HHZ), IR64, Zhonghua11 (ZH11), and Huaidao5 (HD5). Bar = 200 µm. The experiment was repeated twice, and the data are presented as the mean ± standard deviations (pooled from two independent experiments). The different letters indicate significant differences using Duncan's multiple range test (*p* < 0.05).

**Supplementary Figure 3. Chemotaxis of *Meloidogyne graminicola* towards rice root border cells and distribution of serotonergic neurons.** (A) Distribution of serotonergic neurons in *M. graminicola* after 1-hour and 2-hour contact with rice root border cells. MB, median bulb. Bar = 20 µm. (B) Chemotaxis analysis of *M. graminicola* towards different concentrations of root border cells. The experiment was repeated three times. Data are presented as the mean ± standard deviation (calculated from the pooled data of three independent replicates). Different letters indicate significant differences determined by Duncan’s multiple range test.

**Supplementary Figure 4. Morphology of rice root tip and monosaccharide composition of root border cells.** (A) Morphology of a rice root tip section. The section was stained with toluidine blue. Bar = 100 μm. (B) Monosaccharide composition profiles of root border cells from NPB and IR64 varieties. Gal, galactose; Ara, arabinose; Xyl, xylose; Glc, glucose; Fuc, fucose; Man, mannose; Rha, rhamnose; Glu-UA, glucuronic acid; Gal-UA: Galacturonic acid. Data are presented as means ± SD from three independent biological replicates.

**Supplementary Figure 5. Analysis of nematode chemotaxis and aggregation in response to different sugars.** (A) Chemotaxis assay of *Meloidogyne graminicola* toward various sugars. (B) Induction of aggregation behavior in *M. graminicola* by different sugars. The tested sugars include: Xyl (xylose), Ara (arabinose), Gal (galactose), Glc (glucose), Suc (sucrose), Mal (maltose), Cel (cellulose), AG (arabinogalactan), and Pec (pectin). Bar = 500 µm. Data are presented as the mean ± SD (pooled from two independent experiments). Different letters indicate significant differences among treatments using Duncan's multiple range test (*P* < 0.05).

**Supplementary Figure 6. Phylogenetic tree of predicted** **Mg-DEL-1 and Mg-UNC-105 proteins.** Complete amino acid sequences were aligned using ClustalW. The phylogenetic tree was constructed via the Maximum Likelihood method with 1000 bootstrap replicates in MEGA7.0. The Mg-DEL-1 and Mg-UNC-105 of *Meloidogyne graminicola* were highlighted in red.

**Supplementary Figure 7.** **Multiple sequence alignment of Mg-DEL-1 with homologous proteins.** Sequence alignment of Mg-DEL-1 (*Meloidogyne graminicola*), DEL-1 (*Caenorhabditis elegans*, NP_001355359.1) and *Meloidogyne enterolobii* (CAK5091297.1) performed with Clustal X v2.1. Residues highlighted in yellow represent the conserved Amiloride-sensitive sodium channel domain.

**Supplementary Figure 8.** **Multiple sequence alignment of Mg-UNC-105 with homologous proteins.** Sequence alignment of Mg-UNC-105 (*Meloidogyne graminicola*), UNC-105 (*Caenorhabditis elegans*, NP_001254139.1) and *Meloidogyne hapla* (KAL7071408.1) performed with Clustal X v2.1. Residues highlighted in yellow represent the conserved Amiloride-sensitive sodium channel domain.
